# A loss of the cytosolic branched-chain aminotransferase, BCATc, enhances T_h_1 differentiation and skews Tregs to acquire a T_h_1-like phenotype

**DOI:** 10.1097/IN9.0000000000000084

**Published:** 2026-07-13

**Authors:** Tanner J. Wetzel, Christie M. Adam, Tanner J. Kelm, Anna E. Kotula, Madison E. Fagan, Taha Z. Khan, Anthony X. Diehl, Melissa A. Meyer, Jessica P. Sanchez, Brian H. Ladle, Elitsa A. Ananieva

**Affiliations:** 1 Department of Biochemistry and Nutrition, Des Moines University, West Des Moines, IA, USA; 2 Department of Immunology, Microbiology and Pathology, Des Moines University, West Des Moines, IA, USA; 3 Department of Oncology, Sidney Kimmel Comprehensive Cancer Center at Johns Hopkins, Johns Hopkins University, Baltimore, MD, USA

**Keywords:** amino acids, leucine, BCATc, BCATm, T-cell subsets

## Abstract

**Background:**

T-cell fate and function are profoundly shaped by microenvironmental nutrient availability. The branched-chain amino acid, leucine, is essential for T-cell function, with its uptake via the solute carrier family 7-member 5 transporter increasing upon activation. As the enzyme catalyzing the first step of leucine degradation, the cytosolic branched-chain aminotransferase (BCATc) is induced upon T-cell activation and subsequently acts as a negative regulator of T-cell activity. However, a major knowledge gap remains regarding how BCATc modulates T-cell function beyond activation, specifically within differentiated T helper cells.

**Methods:**

Novel T-cell conditional knockout mouse models of BCATc, and its mitochondrial isoenzyme, BCATm, were characterized and used as a source of in vitro or ovalbumin-induced CD4^+^ T-cell subsets in the presence of the leucine competitive antagonist, *N*-acetyl leucine amide (NALA). The studies were complemented by transcriptomic analysis of human T cells using the genomic platform R2.

**Results:**

Loss of BCATc enhanced T_H_1 phenotype as evidenced by the increased expression of T-bet and interferon-*γ*. Leucine, but not the BCAT isoenzymes, was critical for T_H_2 and T_H_17 function as evidenced by the severe reduction in interleukin [IL]-13 and IL-17 release in the presence of NALA. Lastly, a loss of BCATc, or its mitochondrial isozyme, BCATm, facilitated a shift of regulatory T cells (Tregs) to a T_H_1-like phenotype as judged by the increased populations of T-bet^+^Foxp3^+^Tregs.

**Conclusions:**

BCATc is the dominant BCAT isoenzyme with immunoregulatory function in CD4^+^ T-cell subsets and targetable potential in T-cell immunity.

## 1. Introduction

Upon activation, CD4^+^ T cells undergo extensive metabolic reprogramming to enhance nutrient uptake and energy production. In response to the surrounding cytokine milieu, they differentiate into distinct functional subsets, including T_H_1, T_H_2, T_H_17, and regulatory T cells (Tregs) ^[1]^. Akin to their diverse functions, the metabolic demands of CD4^+^ T-cell subsets vary considerably ^[2,3]^. Thus, nutrient availability within the immune landscape may serve as an important signal influencing CD4^+^ T-cell lineage commitment.

Of these nutrients, the branched-chain amino acid (BCAA), leucine, is essential for CD4^+^ T-cell activation and function ^[4–6]^. In addition to serving as a building block for protein synthesis, leucine directly supports metabolic rewiring during CD4^+^ T-cell activation by promoting the activation of complex 1 of the mammalian target of rapamycin mTOR (mTORC1) ^[7]^. Furthermore, failure to supply activated CD4^+^ T cells with sufficient leucine leads to metabolic collapse. This goes beyond mTORC1 regulation, affecting metabolic master regulators, such as the c-Myc oncogene ^[5]^.

Naïve CD4^+^ T cells rely on mitochondrial leucine catabolism to meet basic metabolic needs. This is assisted by the ubiquitously and constitutively expressed BCAT isoenzyme, BCATm. BCATm catalyzes the reversible transamination of leucine to alpha-ketoisocaproic acid (α-KIC) in the mitochondria. Subsequently, α-KIC undergoes irreversible oxidative decarboxylation by the branched-chain α-keto acid dehydrogenase complex, ultimately generating acetoacetate and acetyl-CoA ^[4,8,9]^.

Oppositely, the BCATc isoenzyme is induced upon T-cell receptor engagement in a Nuclear Factor of Activated T cells (NFAT)-dependent manner following CD4^+^ T-cell activation. This is consistent with the role of BCATc in cells and tissues with high metabolic needs, such as the brain, embryonic tissue, and immune cells ^[4,10,11]^.

We have previously shown that BCATc may serve as a negative regulator of CD4^+^ T-cell activation and function. Upon T-cell receptor engagement, leucine entry via the solute carrier family 7-member 5 (Slc7a5) transporter is stimulated, while BCATc expression is induced. By using leucine as a substrate for its transamination reaction, BCATc controls the increased influx of leucine toward mTORC1. However, when CD4^+^ T cells are deficient in BCATc, they show enhanced metabolic capabilities and increased effector function ^[4,5]^.

While previous reports indicate upregulation of BCATc in the tumor microenvironment, few reports have investigated the role of BCATc, or leucine, in nonmalignant CD4^+^ T cells upon lineage commitment ^[7]^. Given this knowledge gap, the objective of this study was to investigate how a loss of BCATc affects CD4^+^ T-cell subset differentiation and function. This was compared with its mitochondrial isoenzyme, BCATm, using both in vitro approaches and in vivo ovalbumin (OVA)-induced mouse models. Additionally, we investigated the impact of leucine antagonism on CD4^+^ T-cell differentiation in cells with normal levels of BCATc/BCATm or when these isoenzymes were deleted.

## 2. Materials and methods

### 2.1 Mice

All mice originated from the C57BL/6 background. Wildtype (WT) mice or mice expressing the CD4-Cre transgene (CD4Cre^0/+^) were purchased from Jackson Laboratories (strain Cat # 017336, Bar Harbor, ME, USA). Mice with loxP-flanked *Bcat1* (T-BCATc^fl/fl^) or *Bcat2* (T-BCATm^fl/fl^) alleles were a gift from S.M. Hutson (Virginia Tech, VA, USA). T-BCATc^fl/fl^ and T-BCATm^fl/fl^ mice were bred with CD4Cre^0/+^ mice to generate mice with a deletion of *Bcat1* (T-BCATc^KO^) or *Bcat2* (T-BCATm^KO^) in single- or double-positive CD4^+^ and CD8^+^ T cells. Genotyping was confirmed on ear genomic DNA with primers (Supplementary Table 1, https://links.lww.com/IN9/A9) to amplify ~330 bp and ~650 bp bands corresponding to the floxed *Bcat1* or *Bcat2* alleles or the *CD4Cre* allele (~300 bp), respectively. All mouse experiments, including the use of anesthesia and euthanasia, were performed according to institutional animal care and use committee-approved protocols from Des Moines University (ID # 2024-06-01, date of approval: June 21, 2024, and ID # 2023-01, date of approval: July 18, 2023) and Johns Hopkins University (protocol date of approval: October 10, 2023). All animal experiments complied with the ARRIVE 2.0 guidelines and were conducted according to the National Institutes of Health guide for the care and use of laboratory animals. The mice were housed in a pathogen-free animal care facility with an alternating 12-hour light/dark cycle and access to food and water ad libitum. Unless specified otherwise, male and female mice, averaged age of 14 ± 3 weeks, were used. Age-matched, T-BCATc^fl/fl^ and T-BCATm^fl/fl^ mice served as littermate controls.

### 2.2 Animal body and organ assessment for indications of inflammation

Organs collected from naïve T-BCATc^KO^ or T-BCATm^KO^ mice and littermate controls (T-BCATc^fl/fl^, T-BCATm^fl/fl^, respectively) were dissected. The gross organ appearance and weights were recorded to monitor for signs of organomegaly. Intestine and liver tissues were screened for genes previously associated with inflammation in mice: chemokine ligand 8 (*Ccl8*), secretory leukocyte protease inhibitor (*Slpi*), tumor necrosis factor alpha (*Tnfa*), and interferon gamma (*Ifng*) ^[12]^. Following total RNA extraction and cDNA synthesis (see procedures below), quantitative reverse transcription polymerase chain reaction (qRT-PCR) was employed to monitor changes in the expression of these genes using primer sequences indicated in Supplementary Table 1, https://links.lww.com/IN9/A9.

### 2.3 In vitro CD4^+^ T-cell isolation and cell culturing

Spleens and lymph nodes from naïve mice were dissected under sterile conditions and pooled to increase T cell yield (*n* = 2–3 spleens/genotype). Spleens and lymph nodes were mechanically disrupted through a nylon stainer to create a single-cell suspension. Lysis with ACK buffer (Cat # A10492-01, Thermo Fisher Scientific, Waltham, MA, USA, spleens only) was performed to remove red blood cells. CD4^+^ T cells were isolated via negative magnetic separation using CD4^+^ T-cell isolation kit (Cat # 130-104-454, Miltenyi Biotec, Bergisch Gladbach, Germany) followed by culture in RPMI-1640 medium (Cat # 10-040-CV, Corning, NY, USA) supplemented with 10% heat inactivated fetal bovine serum (FBS; Cat # 25-011-CV, Corning, New York, NY, USA), 0.1 mg/mL Pen/Strep (Cat # 15140-122, Thermo Fisher Scientific, Waltham, MA, USA), 2 mM Glutamine (Cat # 25030-081, Thermo Fisher Scientific, Waltham, MA, USA), 0.2 mg/mL Gentamycin (Cat # 120-098-661, Quality Biological, Gaithersburg, MD, USA), and 55 μM β-mercaptoethanol (Cat # 21985, Thermo Fisher Scientific, Waltham, MA, USA). Cells were activated with 2 μg/mL plate-bound anti-CD3 (Cat # 40-0031-M001, Cytek Biosciences, Fremont, CA, USA) and 2 μg/mL soluble anti-CD28 (Cat # 40-028-M001, Cytek Biosciences, Fremont, CA, USA). CD4^+^ T cells were seeded at a density of 4 to 5 × 10^6^ cells/well in a six-well plate. Plates were incubated at 37 °C with 5% CO_2_.

### 2.4 In vitro CD4^+^ T-cell differentiation and treatments

CD4^+^ T-cell lineage commitment was induced as previously published ^[13]^ with small modifications. Upon isolation (day 0), CD4^+^ T cells were activated with anti-CD3/anti-CD28 as described earlier and subjected to the following cytokine milieus to induce CD4^+^ T-cell lineage commitment: T_H_1 (interleukin [IL]-12 [10 ng/mL] [Cat # 577002, Biolegend, San Diego, CA, USA], IL-2 [10 ng/mL] [Cat #50-162-8476, Thermo Fisher Scientific, Waltham, MA, USA], and anti-IL-4 [2500 ng/mL] [Cat # 14-7041-85, Thermo Fisher Scientific, Waltham, MA, USA]); T_H_2 (IL-4 [20 ng/Ml] [Cat # AF-2141-42, Thermo Fisher Scientific, Waltham, MA, USA], IL-2 [10 ng/mL], anti-IFN-γ [5000 ng/mL] [Cat # 14-7311-85, Thermo Fisher Scientific, Waltham, MA, USA]); T_H_17 (transforming growth factor [TGF]-β_1_ [20 ng/mL] [Cat # 763104, Biolegend, San Diego, CA, USA], IL-6 [10 ng/mL] [Cat # AF-216-16, Thermo Fisher Scientific, Waltham, MA, USA], anti-interferon [IFN]-γ [5000 ng/mL], and anti-IL-4 [5000 ng/mL]); Treg (TGF-β_1_ [20 ng/mL], IL-2 [10 ng/mL], anti-IFN-γ [5000 ng/mL], and anti-IL-4 [5000 ng/mL]). Activated, but undifferentiated (T_H_0) cells served as controls. Additionally, undifferentiated but activated CD4^+^ T cells were subjected to single cytokine treatments of IL-2 (10 ng/mL), IL-4 (20 ng/mL), IL-6 (10 ng/mL), IL-12 (10 ng/mL), or TGF-β_1_ (20 ng/mL) and compared with untreated but activated cells or untreated/unstimulated cells.

Where indicated, cultures were treated with 100 nM rapamycin (Cat # R-5000, LC Laboratories, Woburn, MA, USA) to target the mTORC1 pathway or 10 mM *N*-acetyl leucine amide (NALA) (Cat # 4000614.0025, Bachem, Bubendorf, Switzerland) to disrupt leucine uptake and metabolism, as previously reported ^[14]^. NALA is a leucine competitive antagonist that consists of l-leucine backbone with an *N*-terminal acetyl group and a C-terminal primary carboxamide (Supplementary Figure 1A, https://links.lww.com/IN9/A8).

For leucine-deprivation medium experiments, a BCAA-free RPMI-1640 medium w/l-glutamine, w/o l-isoleucine, l-leucine, or l-valine (Cat # R8999-20-L, US Biological Life Sciences, Salem, MA, USA) was used. Leucine (50 mg/L, Cat # 61-90-5, Millipore Sigma, Burlington, MA, USA), isoleucine (50 mg/L, Cat # 73-32-5, Millipore Sigma, Burlington, MA, USA), and valine (20 mg/L, Cat # 72-18-4, Millipore Sigma, Burlington, MA, USA) were added back to control T_H_1 and Treg cultures (indicated as leucine “+”). Cells grown in a leucine-free medium (indicated as leucine “−”) were supplemented with isoleucine and valine but not leucine. To avoid a single essential amino acid deprivation, FBS was not dialyzed before addition to the BCAA-free RPMI-1640 medium. The added 10% FBS contributed ~2.4 mg/L-leucine, ~2 mg/L-isoleucine, and ~2.6 mg/L-valine as determined by HPLC analysis ^[15]^.

At 48 hours, media, cytokines, and NALA (where applicable) were replenished at the aforementioned concentrations. At 72 hours, T_H_1, T_H_2, and T_H_17 cells and supernatants were collected. Tregs and supernatants were collected at 96 hours. Cells were stored at −80 °C for qRT-PCR (primers shown in Supplementary Table 1, https://links.lww.com/IN9/A9), or western blotting analysis, or used immediately for cell viability, cell proliferation, or flow cytometry assays.

### 2.5 Cell viability assay

Activated, but undifferentiated, WT CD4^+^ T cells (up to 5 × 10^6^/variant, *n* = 2 independent cultures with *n* = 3 technical replicates) were left untreated or treated with 10 mM NALA or 100 nM rapamycin for 24 hours. At the end of treatment, cells were incubated with 0.5 mg/mL 3-(4,5-dimethylthiazol-2-yl)-2,5-diphenyltetrazolium bromide (Cat # 5224, Tocris Bioscience, Bristol, UK) for 1 hour at 37 °C until 3-(4,5-dimethylthiazol-2-yl)-2,5-diphenyltetrazolium bromide was reduced to an insoluble formazan. Formazan was solubilized with dimethyl sulfoxide, and the absorbance of the resultant purple color was read at 570 and 620 nm using a plate reader. Cell viability was calculated as a percentage of untreated cells.

### 2.6 Cell proliferation assay

WT CD4^+^ T cells (1–2 × 10^6^/variant, *n* = 3 independent cultures with *n* = 3 technical replicates) were isolated as described earlier, washed with sterile 1× phosphate-buffered solution (PBS) buffer, and centrifuged at 1000 rpm for 9 minutes. Cells were labeled with 5 µM carboxyfluorescein succinimidyl ester (Thermo Fisher Scientific, Waltham, MA, USA) in the dark at 37 °C for 15 minutes, and the staining was quenched by adding cold Roswell Park Memorial Institute (RPMI)-1640 medium. The cells were then washed and centrifuged as earlier and left unstimulated or co-stimulated with anti-CD3/anti-CD8 in the absence or the presence of 10 mM NALA for 48 to 72 hours. At the end of each treatment, fluorescence-activated cell sorting analysis (488 nm excitation) was performed to monitor cell proliferation, recorded as discrete peaks on a histogram (undivided, one division, two divisions, etc).

### 2.7 In vivo T-cell stimulation using OVA-producing murine EL4 lymphoma

Murine EG7 (EL4-OVA, Cat # CRL-2113) cells expressing the chicken OVA were purchased from ATCC (Manassas, VA, USA) and maintained in RPMI-1640 medium as described for the CD4^+^ T cells earlier. The heat-inactivated FBS was replaced by regular FBS (Cat # 35-010-CV, Corning, NY, USA), and the OVA antigen was maintained in the presence of 0.04 mg/mL G418 (Cat # 30-234-CR, Corning, NY, USA). Cells, used in their second passage, were free of mycoplasma, and their identity was confirmed by short tandem repeats analysis (LabCorp, Burlington, NC, USA).

T-BCATc^KO^, T-BCATm^KO^ mice, and respective control (T-BCATc^fl/fl^ and T-BCATm^fl/fl^) mice, males and females, age 13 to 19 weeks, were anesthetized via isoflurane and subcutaneously injected with 2.5 × 10^5^ EL4-OVA lymphoma cells in the rear flank. The mice were monitored for 10 days for the appearance of lymphoma tumors. Animal well-being and the general appearance of tumor masses were assessed and subsequently recorded (not shown). On day 10, mice were sacrificed, with tumors, spleens, and draining lymph nodes collected. Of these tissues, spleens and lymph nodes were analyzed further in searching for OVA-induced in vivo changes in splenic and lymphatic T_H_1 and splenic Treg subset populations. For this purpose, spleen and lymph nodes were homogenized as noted above, followed by lysis with ACK buffer to remove red blood cells (spleen only). Single suspensions of splenocytes (5 × 10^6^ cells/well) and cells from lymph nodes (1 × 10^6^ cells/well) were incubated in 1× cell stimulation and protein inhibitor (Golgi plug) cocktail (Cat # 00-4975, Thermo Fisher Scientific, Waltham, MA, USA) for 4 hours in a tissue culture incubator at 37 °C and 5% CO_2._ The cells were next subjected to a fixation/staining procedure to detect T-cell subset-specific transcription factors (T-bet, Foxp3) and IFN-γ as described in detail under Section 2.12. The data were collected using three sets of biological replicates (*n* = 2 mice/set/variant for a total of *n* = 6 mice/variant) with *n* = 3 to 4 internal repeats/biological replicate.

### 2.8 *House dust mite extract experiment to induce T*_*H*_*2 immunity* in vivo

House dust mite (HDM) extract (Dermatophagoides pteronyssinus; Cat # NC9756554, Greer Laboratories, Lenoir, NC, USA) was obtained in lyophilized form and reconstituted in sterile 1× PBS buffer to generate a working stock at 100 µg per 25 µL. T-BCATm^KO^ and T-BCATm^fl/fl^ mice, males and females, age 31 to 35 weeks, *n* = 6 mice/genotype, received intranasal administrations of 100 µg HDM extract or PBS vehicle control once weekly for four consecutive weeks (Supplementary Figure 2A, https://links.lww.com/IN9/A8). Three days after the fourth exposure, mice were euthanized, and bronchoalveolar lavage (BAL) fluid, lung, and spleen tissues were collected. Lung tissue and BAL fluid cell pellets were combined and minced and enzymatically digested for 30 minutes at 37 °C in 5 mL of digest medium (RPMI-1640 with 5% heat inactivated FBS, 0.04 mg/mL DNAse I (Cat # 10104159001, Millipore Sigma, Burlington, MA, USA), 225 units/mL collagenase I (Cat # 17-018-029, Thermo Fisher Scientific, Waltham, MA, USA) and passed through a 70 µM cell filter to obtain a single-cell suspension. Resulting cell suspensions from the lung were prepared for downstream flow cytometric analyses as described under Section 2.12. Gating strategy is shown in Supplementary Figure 3, https://links.lww.com/IN9/A8. Cells were stained with fixable viability dye eFluor 780 (Cat # 65-0865-18, Thermo Fisher Scientific, Waltham, MA, USA). Surface marker–specific antibodies were anti-mouse CD4-BV510 (Cat # 563106, BD Biosciences, Franklin Lakes, NJ, USA), anti-mouse B220-BV650 (Cat # 563893, BD Biosciences, Franklin Lakes, NJ, USA), anti-mouse CD8α-BV786 (Cat # 563332, BD Biosciences, Franklin Lakes, NJ, USA), anti-mouse CD62L-AF488 (Cat # 104420, BioLegend, San Diego, CA, USA), anti-mouse CD44-PerCP (Cat # 103036, BioLegend, San Diego, CA, USA), and anti-mouse CD3-PE-CF594 (Cat # 100246, BioLegend, San Diego, CA, USA). Upon completion of surface staining, cells were fixed and permeabilized using Transcription factor buffer set (Cat # 562574, BD Pharmingen, San Jose, CA, USA) per manufacturer’s recommendation. Intracellular transcription factor staining was performed using anti-mouse GATA3-AF647 (Cat # 653809, BioLegend, San Diego, CA, USA), anti-mouse T-bet-BV605 (Cat # 644817, BioLegend, San Diego, CA, USA), and anti-mouse Foxp3-AF700 (Cat # 56-5773-82, Thermo Fisher Scientific, Waltham, MA, USA). Harvested lung cells were activated ex vivo for 4 hours with phorbol 12-myristate 13-acetate and ionomycin in the presence of protein transport inhibitors brefeldin A and monensin (eBioscience Cell Stimulation Cocktail, Cat # 00-4975-03, San Diego, CA, USA) to induce cytokine production. Intracellular IL-13 (anti-mouse IL-13-PE, Cat # 159403, BioLegend, San Diego, CA, USA) and IFN-γ (anti-mouse IFN-γ-APC, Cat # 554413, BD Biosciences, Franklin Lakes, NJ, USA) were stained using the BD Cytofix/Cytoperm Fixation/Permeabilization Kit (Cat # 554714, BD Biosciences, Franklin Lakes, NJ, USA) according to manufacturer recommendations.

### 2.9 qRT-PCR

mRNA was isolated from whole mouse tissue (ear tissue, intestine, or liver) or cultured CD4^+^ T cells using the Promega SV Total RNA Isolation System Kit (Cat # Z3100, Promega, Fitchburg, WI, USA) according to the manufacturer’s specification. mRNA was quantified using the NanoDrop 8000 (Thermo Fisher Scientific, Waltham, MA, USA). cDNA was synthesized from the isolated mRNA using the Promega GoTaq 2-step qRT-PCR system (Cat # A5001, Promega, Fitchburg, WI, USA) according to the manufacturer’s specification. cDNA was quantified using NanoDrop800. qRT-PCR was performed using the Bio-Rad iTAQ Universal SYBR Green Super Mix (Cat # A25742, Thermo Fisher Scientific, Waltham, MA, USA). Eukaryotic elongation factor one alpha (*Ef1α*) served as an internal control. All primers were created using the NCBI Primer-BLAST tool (https://www.ncbi.nlm.nih.gov/tools/primer-blast/) and purchased from Integrated DNA Technologies. Fold expression changes were calculated using the 2^−ΔΔct^ method. Results were represented as a fold difference of littermate control, or untreated control for NALA treatments. Each measurement was repeated 3 to 4 times, and all independent experiments were averaged as indicated in the figure legends.

### 2.10 Western blotting

Protein was extracted from mouse brain, spleen, or CD4^+^ T-cell pellets, followed by determination of total protein concentration using a microplate BCA assay kit per manufacturer’s specifications (Cat # 23252, Thermo Fisher Scientific, Waltham, MA, USA). Western blotting was performed as previously described ^[4]^. In brief, proteins were separated via SDS-PAGE using 12% polyacrylamide gels and transferred to a PVDF membrane (Cat # IPVH0010, Burlington, MA, USA) using a Bio-Rad Criterion Blotter (Cat # 1704070, Bio-Rad, Hercules, CA, USA). PVDF membranes were blocked in 1× TBST buffer (20 mM Tris base, 136 mM NaCl, 0.1% Tween 20, pH 7.4) containing 4% BSA for 1 hour, followed by the addition of primary antibodies overnight at 4 °C. Anti-BCATc and anti-BCATm ^[16,17]^ were used to detect the protein expression of the cytosolic and mitochondrial BCATs. Antibodies against the eukaryotic translation initiation factor 4E binding protein 1 (4EBP-1, Cat # 9644S), its phosphorylated (P) state, P-4EBP-1 (Thr37/46, Cat # 236B4), the ribosomal protein S6 (S6, Cat # 2217), P-S6 (Ser240/244, Cat # 5364), the AMP-activated protein kinase α (AMPKα, Cat # 2603), P-AMPKα (Thr172, Cat # 2535), and β-tubulin (Cat # 2128), were purchased from Cell Signaling Technology (Denver, MA, USA). After 24 hours, membranes were washed five times in 1× TBST buffer, followed by incubation with a donkey anti-rabbit secondary antibody (Cat # 711-005-152, Immunoresearch Labs, West Grove, PA, USA) for 1 hour before five additional washes. Detection of protein bands was done by incubating the PVDF membranes in a chemiluminescent solution (Cat # 325170, Neogen, Lansing, MI, USA) and by using a developer (Cat # 1170-9-9000, Optimax, Ontario, NY, USA) and X-ray films. The films were photographed, and the bands were quantified using the Image J software ^[18]^. Protein bands corresponding to BCATc and BCATm were normalized to β-tubulin as a loading control. Protein bands corresponding to P-AMPK, AMPK, P-4EBP-1, 4EBP-1, P-S6, and S6 were represented as the ratio between the phosphorylated and total concentrations of AMPK, 4EBP-1, and S6, respectively. Results were represented as a fold change of unstimulated control, or as a percentage of undifferentiated or untreated CD4^+^ T cells. Each sample was loaded on a protein gel between 2 and 3 times, and representative protein blot images are shown as indicated in the figure legends.

### 2.11 Enzyme-Linked Immunosorbent Assay

Following in vitro differentiation of CD4^+^ T cells, supernatants were collected, and secretion of the following cytokines was measured via Enzyme-Linked Immunosorbent Assay (ELISA) per manufacturer’s recommendation: IL-10 (Cat # 88-7105-22, Thermo Fisher Scientific, Waltham, MA, USA), IFN-γ (Cat # 88-7314, Thermo Fisher Scientific, Waltham, MA, USA), IL-13 (Cat # 88-7137-22, Thermo Fisher Scientific, Waltham, MA, USA). and IL-17 (Cat # 88-7371-22, Thermo Fisher Scientific, Waltham, MA, USA). Results were standardized to 1 × 10^6^ cells and were represented as picograms (pg) of secreted cytokine per 1 × 10^6^ cells. Each measurement was repeated 3 to 6 times, and a single representative experiment is shown.

### 2.12 Flow cytometry

Single-cell suspensions from spleens, thymuses, lymph nodes, and lungs were prepared as described earlier. Cell counts of single-cell suspensions were determined using a hemocytometer and trypan blue exclusion dye and washed in 1× stain buffer (Cat # 554656, BD Pharmingen, San Jose, CA, USA) before use. For detection of CD4^+^ T-cell subset extracellular markers (spleen, lymph nodes, or thymus), between 1 and 5 × 10^6^ cells/sample were incubated with fluorescently conjugated antibodies against CXCR3 (T_H_1), CD294 (T_H_2), CCR6 (T_H_17), and CD25 (Tregs) or corresponding isotype control antibodies in 1× stain buffer for 30 minutes on ice in the dark. Upon completion of surface staining, cells were fixed and permeabilized using Transcription Factor Buffer Set (Cat #562574, BD Pharmingen, San Jose, CA, USA) per manufacturer’s recommendation. Intracellular staining was performed with fluorescent antibodies against T-bet (T_H_1), GATA3 (T_H_2), RORγt (T_H_17), or Foxp3 (Tregs), or respective isotype control antibodies. Flow cytometry was performed using the Attune NxT acoustic focusing flow cytometer (Thermo Fisher Scientific, Waltham, MA, USA) with blue (488 nm) and red (637 nm) lasers or BD FACSCelesta (BD Biosciences) flow cytometer configured with blue, red, and violet (405 nm) lasers. Data analysis was performed using accompanying Attune cytometric v6.2.1 or FlowJo v10.10.0 software. The gating strategy used to identify T-cell subtypes in spleens, lymph nodes, or thymus can be viewed in Supplementary Figure 4, https://links.lww.com/IN9/A8. The following antibodies were used: anti-CD4-Alexaflour488 (Cat # 51-0041-82, Thermo Fisher, Waltham, MA, USA), rat IgG2b kappa-Alexaflour488 (Cat # 53-4031-80, Thermo Fisher Scientific, Waltham, MA, USA), anti-CD8-PE-Cy7 (Cat # A15385, Thermo Fisher Scientific, Waltham, MA, USA), rat IgG2a kappa-PE-Cy7 (Cat # 25-4321-82, Thermo Fisher Scientific, Waltham, MA, USA), anti-CD25-APC (Cat # BDB5571 92, BD Biosciences, Franklin Lakes, NJ, USA), rat IgG1 lambda—APC (Cat # BDB550884, BD Biosciences, Franklin Lakes, NJ, USA), anti-Foxp3-PE (Cat # BDB566881, BD Biosciences, Franklin Lakes, NJ, USA), mouse IgG1 kappa-PE (Cat # BDB554680, BD Biosciences, Lakes, NJ, USA), anti-CXCR3-APC (Cat # 17-1831-82, Thermo Fisher Scientific, Waltham, MA, USA), Armenian hamster IgG control-APC (Cat # 17-4888-82, Thermo Fisher Scientific, Waltham, MA, USA), anti-T-bet-PE (Cat # 12-5825-82, Thermo Fisher Scientific, Waltham, MA, USA), mouse IgG1 kappa-PE (Cat # BDB554680, BD Biosciences, Lakes, NJ, USA), anti-T-bet-PE-Cy7 (Cat # 25-5825-82, Thermo Fisher Scientific, Waltham, MA, USA), mouse IgG1 kappa-PE-Cy7, anti-T-bet- PerCP-Cyanine5.5 (Cat # 45-5825-82, Thermo Fisher Scientific, Waltham, MA, USA), mouse IgG1 kappa-PerCP-Cyanine5.5 (Cat # 45-4714-82, Thermo Fisher Scientific, Waltham, MA, USA), anti-CD294-APC (Cat # 51-2941-82, Thermo Fisher Scientific, Waltham, MA, USA), rat IgG2a kappa-Alexaflour647 (Cat # 51-4321-81, Thermo Fisher Scientific, Waltham, MA, USA), anti-GATA3-PE (Cat # 12-9966-42, Thermo Fisher Scientific, Waltham, MA, USA), mouse IgG1 kappa-PE (Cat # BDB554680, BD Biosciences, Franklin Lakes, NJ, USA), anti-CD161-APC (Cat # 17-1619-42, Thermo Fisher Scientific, Waltham, MA, USA), anti-IL-13-APC-efluor780 (Cat # 47-7133-82, Thermo Fisher Scientific, Waltham, MA, USA), anti-ROR*γ*t-PE (Cat # 12-6988-82, Thermo Fisher Scientific, Waltham, MA, USA), anti-IFN-*γ*-PE (Cat # 12-7311-41, Thermo Fisher Scientific, Waltham, MA, USA), and rat IgG2a kappa- PE (Cat # 12-4321-80, Thermo Fisher Scientific, Waltham, MA, USA).

### 2.13 Human gene expression datasets acquisition and analysis

Human RNA expression datasets were accessed using the genomics and visualization platform (R2) (http://r2.amc.nl). R2 provided genomic information to analyze the differential expression of *BCAT1* (the gene encoding BCATc) and compare it to that of *BCAT2* (the gene encoding BCATm) in immune cells from healthy donors and patients infected with the hepatitis C virus (HCV) or diagnosed with rheumatoid arthritis (RA) or systemic lupus erythematosus (SLE). The HCV dataset consisted of 15 randomized and mixed-sex-derived samples (healthy control (*n* = 5/cell type), HCV, combined low and high load (*n* = 10/cell type) ^[19]^. The RA/SLE dataset, deposited by Lauwerys et al (https://www.ncbi.nlm.nih.gov/geo/query/acc.cgi?acc=GSE4588), included specimens derived from PBMCs of healthy donors (CD4^+^ T cells, *n* = 10; B cells, *n* = 9) and patients with RA (CD4^+^ T cells, *n* = 8; B cells, *n* = 7) and SLE (CD4^+^ T cells, *n* = 8; B cells, *n* = 7). Patients’ characteristics were not specified in this report.

All data were extracted from Affymetrix gene chip human genome U133 plus 2.0 arrays embedded in the R2 platform and transformed into the Log2 scale, averaged, and subjected to statistical analysis as explained under “Statistics.” Specimens from RA and SLE patients were analyzed in a single combined group labeled as the “disease state”, or “D”. Differential gene expression analysis of *BCAT1* or *BCAT2* was performed using the “singular gene versus track” analysis in the R2 platform. Correlation between the gene expression of *BCAT1* or *2* and major subset transcription factors *TBX21, IFNG, STAT4* (T_H_1), *GATA3, IL4* (T_H_2), *FOXP3, TGFB1, LGALS3* (Tregs), *RORC, RORA, IL17A* (T_H_17) was performed using the “correlate 2 gene” analysis and calculated utilizing the Pearson coefficient (*R*) function. GraphPad was used to generate heatmaps of the data.

### 2.14 KEGG pathway analysis

The R2 platform (http://r2.amc.nl) was used to screen publicly available gene expression profiles originating from CD4^+^ T-cell subsets of healthy human donors. The search identified only one study with gene expression profiles obtained from activated Tregs (*n* = 5) or T helper (*n* = 5) cells isolated from tonsils of healthy human donors ^[20]^. The KEGG pathway analysis of *BCAT1* and *BCAT2* for each subset track (Treg or T helper cells) was directly generated in the R2 platform with the following specifications: the “KEGG pathway finder for gene correlation” analysis was selected, followed by identifying the gene reporter (*BCAT1* or *BCAT2*) using transformation Log2, and correlation *R* that included positive and negative gene correlations and correlation *P* value cutoff <0.01. The first ten KEGG pathways, and associated genes, were extracted and presented with schematics generated in GraphPad. Genes that significantly correlated with *BCAT1* or *BCAT2,* but appeared associated with more than one KEGG pathway, are presented in bold.

### 2.15 Statistics

A two-tailed Student’s *t*-test was used to compare T-BCATc^KO^ and T-BCATm^KO^ mice with respective age-matched control T-BCATc^fl/fl^ and T-BCATm^fl/fl^ mice using GraphPad Prism v10. For T-cell in vitro experiments, cells were pooled from *n* = 3 to 5 mice/genotype/experiment to ensure adequate T-cell material. For OVA-induced T-cell in vivo experiments, spleens were pooled from *n* = 2 mice/genotype/experiment for a total of *n* = 6 mice. The majority of the experiments were repeated three independent times with *n* = 3 to 6 internal repeats per biological replicate (eg, a single mouse or pooled mice) and averaged (Ave + standard error of the mean [SEM]) or shown as a single representative experiment (Ave ± standard deviation) as indicated in the figure legends. Experiments involved both male and female mice. Although each T-cell conditional mouse line was initially analyzed by sex, data from downstream in vitro and in vivo assays were pooled because no apparent sex differences were observed (indicated as mixed sex). *P < 0.05, **P < 0.01, or ***P < 0.001 was considered statistically significant.

### 2.16 Ethical approval

All mouse experiments, including the use of anesthesia and euthanasia, were performed according to institutional animal care and use committee-approved protocols from Des Moines University (ID # 2024-06-01, date of approval: June 21, 2024, and ID # 2023-01, date of approval: July 18, 2023) and Johns Hopkins University (protocol date of approval: October 10, 2023). All animal experiments complied with the ARRIVE 2.0 guidelines and were conducted according to the National Institutes of Health guide for the care and use of laboratory animals.

## 3. Results

### 3.1 BCATc and its mitochondrial isoenzyme, BCATm, are differentially regulated during CD4^+^ T-cell lineage commitment

To investigate the role of BCATc in CD4^+^ T-cell differentiation, WT CD4^+^ T cells were isolated and in vitro differentiated into iT_H_1, iT_H_2, iT_H_17, or iTregs (indicated with “i” for inducible T-cell subsets). Successful lineage commitment was verified by the increased gene/protein expression of lineage transcription factors *Tbx21/*T-bet (T_H_1), *Gata3*/GATA3 (T_H_2), *Rorc*/RORγt (T_H_17), and *Foxp3*/Foxp3 (Tregs) and major lineage cytokines IFN-γ (T_H_1), IL-13 (T_H_2), IL-17 (T_H_17), and IL-10 (Tregs) (Supplementary Figure 5A–D https://links.lww.com/IN9/A8).

**Figure 1.**
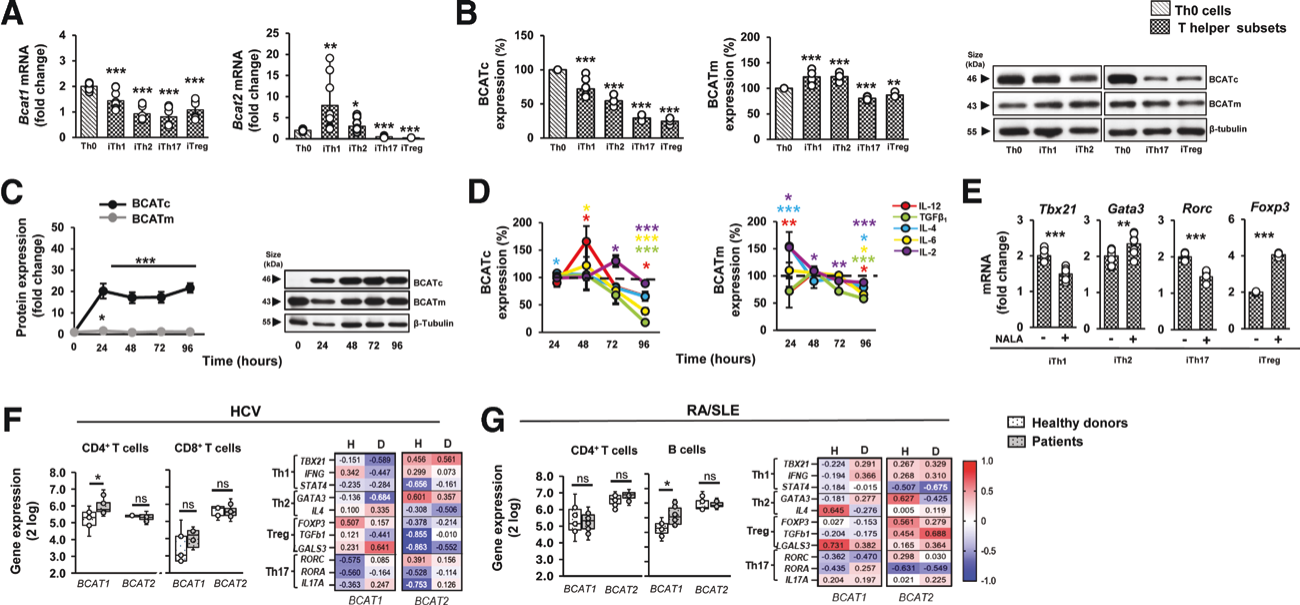
**Characterization of BCATc and BCATm in differentiated CD4^+^T cells.** (A–E) CD4^+^ T cells, isolated from spleens and lymph nodes of WT mice, were activated and treated with skewing cytokines to compare the expression of BCATc and BCATm upon subset differentiation. (A) *Bcat1* and *Bcat2* mRNA (*n* = 9 mice/variant) and (B) BCATc and BCATm protein levels (*n* = 6–9 mice/variant) in different T-cell subsets. (C) Time course of BCATc and BCATm expression in activated but undifferentiated cells (*n* = 12 mice). (D) Time course of BCATc and BCATm expression in activated cells in the absence or the presence of individual T-cell skewing cytokines (*n* = 5–12 mice/cytokine/time point). (E) mRNA levels of T-cell lineage transcription factors in activated and differentiated cells treated with 10 mM NALA (*n* = 6–9 mice/variant). (F, G) Comparison of *BCAT1* and *BCAT2* expression between lymphocytes from healthy (H) human donors and patients under disease (“D”) state. (F) *BCAT1* and *BCAT2* expression in CD4^+^ and CD8^+^ T cells from healthy donors (*n* = 5 donors/cell type) and patients with HCV (*n* = 10 patients/cell type), accompanied by a heat map showing the *R* values of *BCAT1* or *BCAT2* correlation with respective T-cell lineage transcription factors. (G) *BCAT1* and *BCAT2* expression in CD4^+^ T cells (*n* = 10) and B cells (*n* = 9) from healthy donors and patients with RA/SLE (*n* = 14–16 patients/cell type), accompanied by a heat map showing the *R* values of *BCAT1* or *BCAT2* correlation with respective T-cell lineage transcription factors. In A–E panels, data represent 2 to 3 independent experiments with *n* = 3 to 6 pooled mouse spleens and lymph nodes/experiment, mixed sex. The Western blot images are representative of three independent experiments. Average ± SEM. Statistical significance as determined by a two-tailed Student’s *t*-test: *P < 0.05, **P < 0.01, ***P < 0.001.

Following CD4^+^ T-cell differentiation, *Bcat1* (the mouse gene for BCATc) was significantly downregulated across all subsets compared with activated T_H_0 cells. Conversely, the expression of *Bcat2* (the mouse gene for BCATm) was reduced in iT_H_17 and iTregs but increased in iT_H_1 and iT_H_2 cells (Figure 1A). These changes were confirmed at the protein level (Figure 1B).

With varying time courses in our subset differentiation protocol, we asked if the alterations in BCATc and BCATm expression were due to time-dependent effects post-T-cell activation. WT CD4^+^ T cells were activated for 24 to 96 hours to monitor for time-dependent changes in BCATc or BCATm. Aligning with previous observation ^[4]^, BCATc was significantly upregulated by ~20-fold upon CD4^+^ T-cell activation in contrast to unstimulated T cells that do not express BCATc. BCATc remained elevated at 96 hours, with no significant differences between 24 and 96 hours (Figure 1C). As expected, BCATm was constitutively expressed, and although its expression was mildly elevated at 24 hours post-activation (~1.5-fold), the activated CD4^+^ T cells expressed BCATm at levels similar to those of the unstimulated T cells (Figure 1C). These results suggest that activated T cells maintain the expression of BCAT isoenzymes independent of time-limiting differentiation conditions.

Further, we asked if the observed changes in BCATc and BCATm expression were due to the influence of individual lineage-skewing cytokines. WT CD4^*+*^ T cells were activated and simultaneously treated with one of the major skewing cytokines for 24 to 96 hours as follows: IL-2 or IL-12 (T_H_1), IL-4 (T_H_2), IL-6 or TGF-β_1_ (T_H_17), and TGF-β_1_ or IL-2 (Tregs). Despite initial fluctuations during early activation, the BCATc expression was significantly reduced at 72 and 96 hours, a pattern akin to that seen in fully differentiated CD4^+^ T cells (Figure 1D and compare with Figure 1B). BCATm expression was reduced at 72 and 96 hours, but to a lesser extent than BCATc (Figure 1D).

Given the essential role of leucine for T-cell activation and function ^[5]^, we next investigated if leucine alone influences CD4^+^ T-cell differentiation. To induce leucine restriction, we treated the cells with 10 mM of the leucine structural antagonist NALA, a concentration previously shown effective in mouse CD4^+^ T cells, Jurkat T cells, and human cancer cell lines ^[14,21,22]^. Before differentiation, we verified that 10 mM NALA caused only a mild 13% reduction in activated CD4^+^ T-cell viability, which is substantially less toxic than the 56% inhibition observed with 100 nM rapamycin (Supplementary Figure 1B https://links.lww.com/IN9/A8). Treatment with 10 mM NALA significantly reduced cell proliferation by 16.7% at 48 hours and 41% at 72 hours (Supplementary Figure 1C,D, https://links.lww.com/IN9/A8). These results were expected as they were consistent with NALA inhibition of mTORC1 signaling in activated CD4^+^ T cells and Jurkat T cells ^[14,22]^.

Results with differentiated T cells demonstrated that iT_H_1 and iT_H_17 cells expressed significantly less *Tbx21* and *Rorc* in the presence of 10 mM NALA (Figure 1E). Oppositely, NALA enhanced the expression of *Gata3* and *Foxp3* in iT_h_2 and iTregs, respectively, when compared with differentiated but untreated cells (Figure 1E). Overall, the results suggest that T cells rely on leucine and its transamination by the BCAT isoenzymes in a subset-dependent manner. This points toward a unique metabolic needs of each subset.

Lastly, we used the publicly accessible R2 genomics and visualization platform to compare *BCAT1* and *BCAT2* in CD4^+^ T cells from healthy human donors and those isolated from patients diagnosed with HCV, RA, or SLE. *BCAT1,* but not *BCAT2*, was significantly upregulated in CD4^+^ T cells from patients with HCV but not in patients with RA or SLE (combined under disease state, “D”) (Figure 1F,G). Interestingly, *BCAT1* was also upregulated in B cells of patients suffering from RA or SLE (Figure 1G). Correlation analysis between *BCAT1* or *BCAT2* and major subset transcription factors, under healthy and disease states, further revealed a negative correlation between *BCAT1* and *TBX21*, *STAT4*, *GATA3*, *RORC*, or *RORA*, while *BCAT2* and *TBX21*, *GATA3*, or *RORC* were positively correlated in CD4^+^ T cells of healthy donors (Figure 1F,G). Under disease state, however, these correlations fluctuated substantially, suggesting that *BCAT1* and *BCAT2* may be subjected to differential regulations and may play noncanonical roles across disease states (Figure 1F,G).

### 3.2 Deletion of BCATc or BCATm in newly developed T-cell conditional mouse models does not trigger inflammation or alter T-cell populations in naïve mice

**Figure 2.**
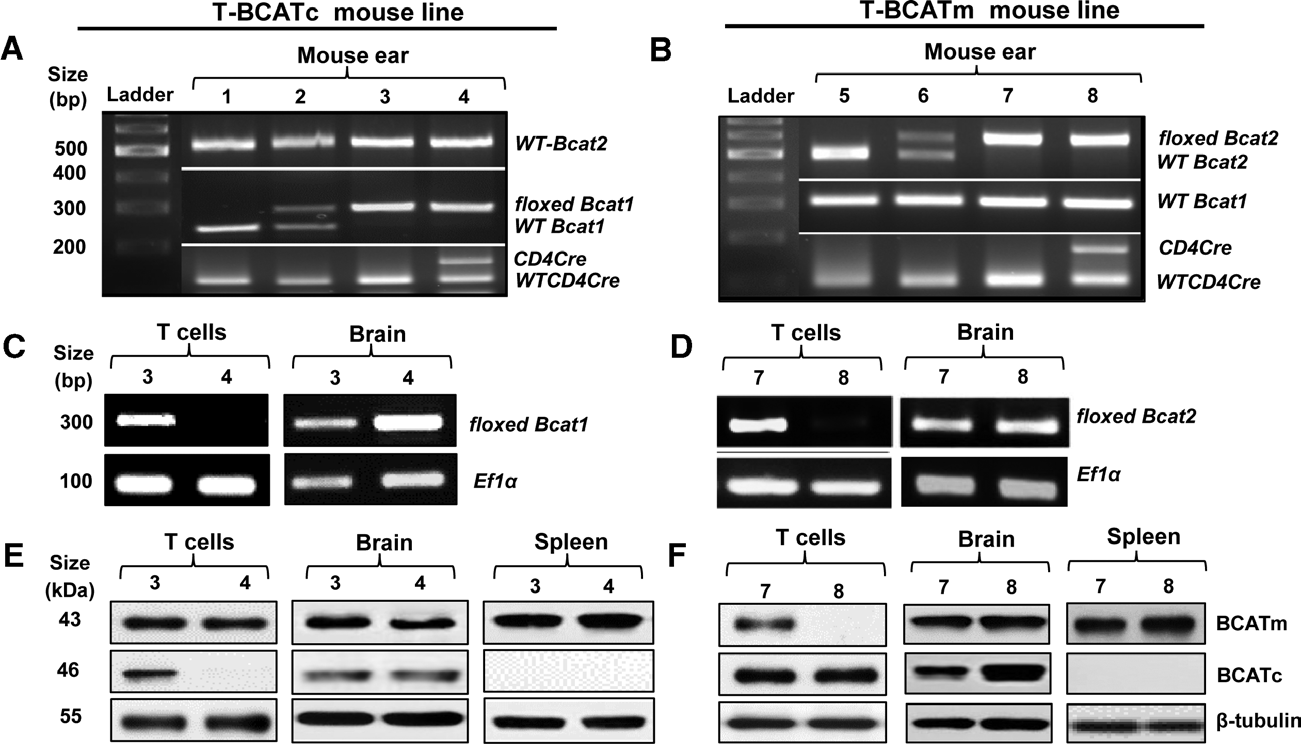
**Characterization of mice carrying a single deletion of *Bcat1 or Bcat2 in* T cells.** (A, B) Genomic DNA from mouse ear showing presence of floxed (fl) alleles of *Bcat1* (encodes BCATc, refer to “3” and “4”) or *Bcat2* (encodes BCATm, refer to “7” and “8”). The *CD4Cre* allele in “4” and “8” was maintained in a hemizygous state. A separate WT band was amplified to confirm this state (*WTCD4Cre*). In addition, “4” and “8” contained the WT alleles of *Bcat1* or *Bcat2*, respectively. (C, D) cDNA produced by a reverse transcriptase, confirming the presence of floxed *Bcat1 or Bcat2* in CD4^+^ T cells (“3” and “7”) or brain tissues (“3-4”,”7-8”, positive control). In contrast, floxed *Bcat1* or *Bcat2* transcripts were absent from activated CD4^+^ T cells isolated from T-BCATc^KO^ or T-BCATm^KO^ mice, respectively (refer to “4” and “8” in C, D). Eukaryotic translation elongation factor (*EF1α*) was used as a loading control. (E, F) Western blotting confirming the loss of expression of BCATc (“4”) or BCATm (“8”) from activated CD4^+^ T cells and spleen (negative control for BCATc), but not brain (positive control). β-Tubulin was used as a loading control. CD4^+^ T cells were activated as described under Methods. In all panels, at least three independent experiments (*n* = 3–6 mice/variant/experiment, mixed sex) were performed. The images are representative of DNA or protein gels of tissue-specific samples. Variant names and genotypes: “1” and “5” is WT mouse [*Bcat1*^*+/+*^*Bcat2*^*+/+*^ CD4*Cre*^-^], “2” is Heterozygous mouse by BCATc [*Bcat1*^*fl/+*^*Bcat2*^*+/+*^CD4*Cre*^-^] or “6” by BCATm [*Bcat1*^*+/+*^*Bcat2*^*fl/+*^CD4*Cre*^-^], “3” is T-BCATc^fl/fl^ mouse [*Bcat1*^*fl/fl*^*Bcat2*^*+/+*^CD4*Cre*^-^], “7” is T-BCATm^fl/fl^ mouse [*Bcat1*^*+/+*^*Bcat2*^*fl/fl*^CD4*Cre*^-^], “4” is T-BCATc^KO^ mouse [*Bcat1*^*fl/fl*^*Bcat2*^*+/+*^CD4*Cre*^*+/0*^] with T cell: [*Bcat1*^*−/−*^*Bcat2*^*+/+*^CD4*Cre*^*+/0*^] and “8” is T-BCATm^KO^ mouse [*Bcat1*^*+/+*^*Bcat2*^*fl/fl*^CD4*Cre*^*+/0*^] with T cell: [*Bcat1*^*+/+*^*Bcat2*^*−/−*^CD4*Cre*^*+/0*^].

We created single conditional knockouts of *Bcat1* and *Bcat2* in mouse T cells to compare the effect of the loss of each isoenzyme on CD4^*+*^ T-cell subset differentiation and function. CD4Cre-mediated recombination was used to remove the floxed *Bcat1* or *Bcat2* from double-positive (CD4^+^CD8^+^) and single-positive CD4^+^ and CD8^+^ T cells of the offspring of T-BCATc^KO^ and T-BCATm^KO^ mice (“4” and “8” in Figure 2A,B, respectively). A loss of cDNA from T cells, but not brain cDNA (positive control), obtained from reverse transcriptase reactions, verified the deletion of *Bcat1* and *Bcat2* from T cells of T-BCATc^KO^ and T-BCATm^KO^ mice (“4” and “8” in Figure 2C,D). Western blotting confirmed the loss of BCATc or BCATm in isolated T cells (“4” and “8” in Figure 2E,F). Normal systemic expression levels of the floxed versions of BCATc and BCATm remained unchanged (“3” and “7” in Figure 2E,F).

**Figure 3.**
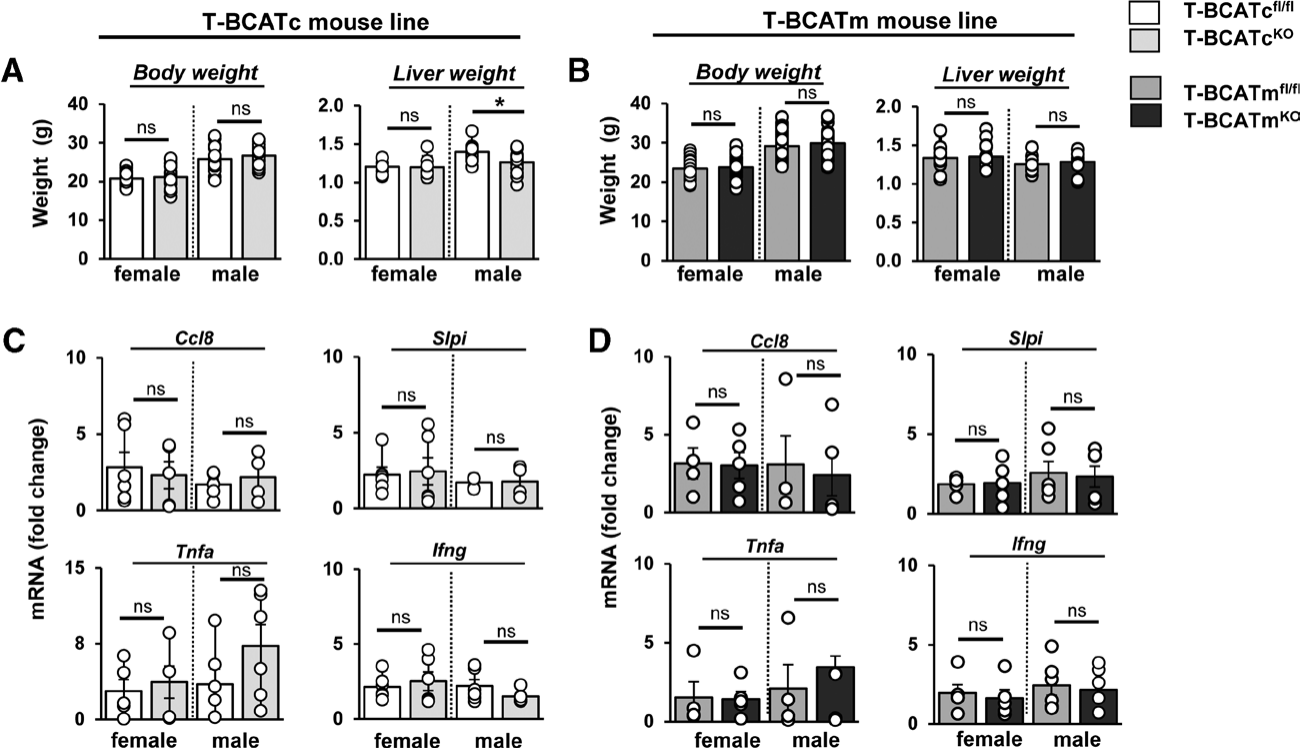
**A loss of *Bcat1 or Bcat2* expression from mouse T cells does not manifest in an inflammatory phenotype in the liver.** Total body and liver weights (A,B) and mRNA expression of liver inflammatory markers (C,D) were measured in naïve T-BCATc^KO^ and T-BCATm^KO^ mice, as compared to age-matched control T-BCATc^fl/fl^ and T-BCATm^fl/fl^ mice, respectively. Liver weights were standardized to 25 g body weight. In all panels, data represent average ± SEM, *n* = 12 mice/genotype, separated by sex (*n* = 6/sex). In C and D, each sample was run in 3 to 4 internal repeats. Statistical significance as determined by a two-tailed Student’s *t*-test: *P < 0.05, ns: no significant difference. *Ccl8:* chemokine ligand 8; *Slpi*: secretory leukocyte protease inhibitor; *Tnfa*: tumor necrosis factor alpha; *Ifng*: interferon gamma.

Given the transgenic modulation of the T cells, mice from each colony were assessed for macroscopic and molecular indications of inflammation. Compared with T-BCATc^fl/fl^ and T-BCATm^fl/fl^ control mice, there was no difference in total body weight (Figure 3A,B) or indications of organomegaly in the heart, lungs, brain, thymus, kidneys, or spleens of T-BCATc^KO^ or T-BCATm^KO^ mice, regardless of sex. Male T-BCATm^KO^ mice had a small reduction in spleen size when compared to male T-BCATm^fl/fl^ mice (Supplementary Table 2, https://links.lww.com/IN9/A9).

Organ-specific analysis of the liver revealed male T-BCATc^KO^ mice had significantly smaller livers compared to those from T-BCATc^fl/fl^ control mice (Figure 3A). To assess if the reduction in liver weight was caused by an inflammatory response, liver tissue was screened for changes in genes associated with inflammation, such as *Ccl8*, *Slpi*, *Tnfa*, or *Ifng.* The expression of these genes was unchanged upon BCATc or BCATm deletion, regardless of sex (Figure 3C,D). Likewise, analysis of intestines and colons revealed no changes in total weight and lengths or in the tested inflammatory markers between animal groups (Supplementary Figure 6, https://links.lww.com/IN9/A8). Naïve T-BCATc^KO^ and T-BCATm^KO^ mice did not show significant changes in total counts of splenic, lymphatic, or thymic CD4^+^ T cells (Supplementary Figure 7A–C, https://links.lww.com/IN9/A8). Similarly, the counts of splenic or lymphatic T_H_1, T_H_17, Treg populations (Supplementary Figure 8A–F, https://links.lww.com/IN9/A8), or thymic T_H_2 populations (Supplementary Figure 8G, https://links.lww.com/IN9/A8), were not statistically different from those of T-BCATc^fl/fl^ and T-BCATm^fl/fl^ mice, respectively. Overall, these results indicate that deletion of BCATc or BCATm from CD4^+^ T cells does not impact the localization or numbers of CD4^+^ T-cell subsets and does not induce inflammation in naïve C57BL/6 mice.

### 3.3 *Loss of BCATc, but not BCATm, promotes T*_*H*_*1 phenotype and increases IFN-γ* *secretion*

**Figure 4.**
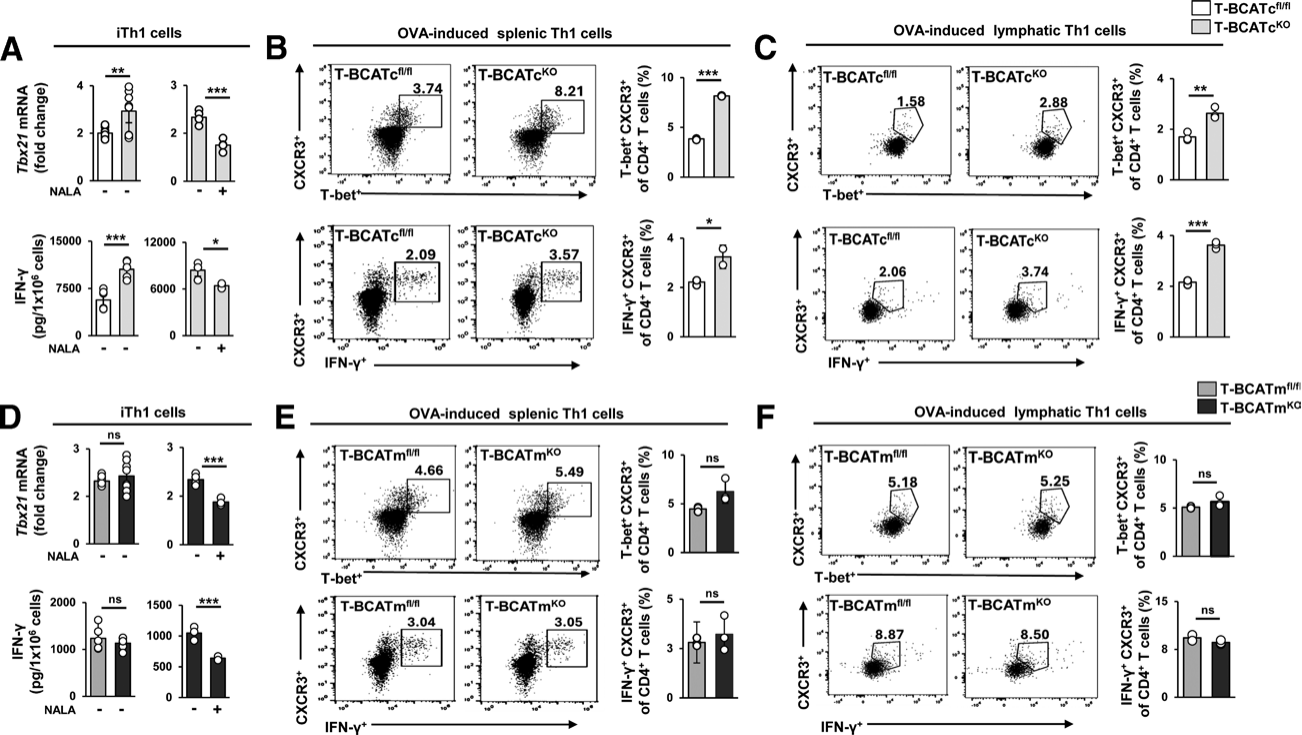
**BCATc, but not BCATm, enhances T**_**H**_**1 subset differentiation and function.** Mice from respective groups were either used as a source of CD4^+^ T cells for in vitro differentiation into iT_H_1 cells or were challenged with OVA-producing EL4 mouse lymphoma cells for 10 days to induce T_H_1 immune response in vivo. (A–C) Results obtained using T-BCATc^KO^ and T-BCATc^fl/fl^ mice. (A) *Tbx21* mRNA expression and secretion of IFN-γ from splenic/lymphatic iT_H_1 cells in the absence or the presence of 10 mM NALA (*n* = 3–10 mice/variant). (B,C) Representative flow charts along with average bar graphs showing T-bet expression and IFN-γ production in OVA-induced splenic (B) and lymphatic (C) T_H_1 (CXCR3^+^CD4^+^) cells (*n* = 6 mice/variant, a representative experiment is shown). (D–F) Results obtained using T-BCATm^KO^ and T-BCATm^fl/fl^ mice. (D) *Tbx21* mRNA expression and secretion of IFN-γ from splenic/lymphatic iT_H_1 cells in the absence or the presence of 10 mM NALA (*n* = 3–6 mice/variant). (E,F) Representative flow charts along with average bar graphs showing T-bet expression and IFN-γ production in OVA-induced splenic (E) and lymphatic (F) T_H_1 (CXCR3^+^CD4^+^) cells (*n* = 6 mice/variant, a representative experiment is shown). For all panels, average ± SEM or ± SD of mixed sex. Statistical significance as determined by a two-tailed Student’s *t*-test: *P < 0.05, **P < 0.01, ***P < 0.001, or ns = no significance.

The individual impact of BCATc on CD4^+^ T cell differentiation and function was explored by skewing T cells toward the iT_H_1 subset in vitro. Alternatively, this impact was examined by inducing T_H_1 subset differentiation in spleen and lymph nodes following in vivo stimulation of T-BCATc^fl/fl^ and T-BCATc^KO^ mice with OVA-producing murine EL4 lymphoma (Figure 4A–C).

iT_H_1 cells from T-BCATc^KO^ mice exhibited significantly higher *Tbx21* expression (46% increase) and IFN-γ secretion (86% increase) compared with iT_H_1 cells from T-BCATc^fl/fl^ mice (Figure 4A). Notably, treating T-BCATc^KO^ iT_H_1 cells with NALA reversed these effects, lowering *Tbx21* expression by 44% and IFN-γ release by 24%, respectively (Figure 4A). OVA-induced T_H_1 responses in T-BCATc^KO^ mice revealed that BCATc deficiency correlated with increased T-bet^+^CXCR3^+^CD4^+^ T cell populations in both the spleen (1.1-fold increase) and lymph nodes (55% increase) compared to controls (Figure 4B,C). Furthermore, these cells produced higher levels of IFN-γ in the spleen (46%) and lymph nodes (68%) of T-BCATc^KO^ mice upon OVA stimulation (Figure 4B,C). In contrast, the loss of the mitochondrial isoenzyme, BCATm, did not significantly alter T-bet expression or IFN-γ release, either in vitro or upon OVA stimulation of T-BCATm^KO^ mice (Figure 4D–F). Addition of NALA to iT_H_1 cells of T-BCATm^KO^ mice reduced *Tbx21* mRNA and IFN-γ secretion in a similar manner as shown for the T-BCATc^KO^ mice (compare NALA in Figure 4A,D). To elucidate the mechanism behind the differential T_H_1 responses to BCAT isoenzymes, we analyzed the phosphorylation of the mTORC1 targets S6 and 4EBP-1. Loss of BCATc significantly increased S6 (~59%) and 4EBP-1 (~52%) phosphorylation in complete RPMI-1640 medium (Supplementary Figure 9A, https://links.lww.com/IN9/A8). However, when BCATc-deficient iT_H_1 cells were cultured in leucine-free medium, 4EBP-1 phosphorylation dropped by approximately 51%, while S6 phosphorylation remained unaffected (Supplementary Figure 9A, https://links.lww.com/IN9/A8). This suggests that the impact of BCATc on T_H_1 cells is, at least in part, mediated through the mTORC1 pathway. Unlike BCATc, the loss of BCATm in iT_H_1 cells caused a significant ~91% increase in S6 phosphorylation but failed to activate 4EBP-1. In fact, 4EBP-1 phosphorylation was reduced by ~41% (Supplementary Figure 9B, https://links.lww.com/IN9/A8). These findings demonstrate that BCATm selectively affects the S6 and 4EBP-1 branches of mTORC1 pathway, explaining at least in part the minimal impact of BCATm deficiency on T_H_1 differentiation and function.

### 3.4 *Leucine restriction reduces T*_*H*_*  2 and T*_*H*_*17 functionality, whereas disrupting leucine degradation only partially affects them*

**Figure 5.**
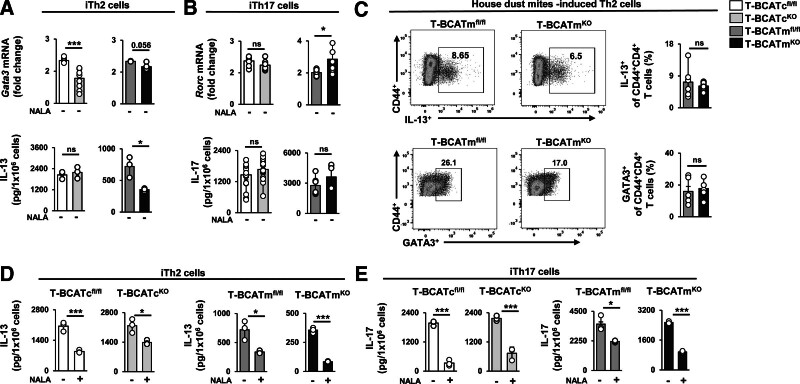
**T**_**H**_**2 and T**_**H**_**17 function is dependent on leucine but not leucine degradation.** Mice from respective groups were either used as a source of CD4^+^ T cells for in vitro differentiation into iT_H_2 and iT_H_17 cells or were challenged with house dust mites (HDM, T-BCATm^KO^, and control mice only) for 4 weeks to induce T_H_2 immune response in vivo. (A) *Gata3* mRNA expression and secretion of IL-13 from iT_H_2 cells. T-BCATc mouse colony, *n* = 6–9 mice/variant, T-BCATm mouse colony, *n* = 3 mice/variant. (B) *Rorc* mRNA expression and secretion of IL-17 from iT_H_17 cells. T-BCATc mouse colony, *n* = 6 mice/variant, T-BCATm mouse colony, *n* = 3–6 mice/variant. (C) Representative flow charts along with average bar graphs showing IL-13 production and GATA3 expression by CD44^+^CD4^+^ T cells isolated from the lungs of HDM sensitized T-BCATm^KO^ and littermate control T-BCATm^fl/fl^ mice (*n* = 6 mice/variant). (D-E) IL-13 and IL-17 secretion from iT_H_2 and iT_H_17 cells in the absence or the presence of 10 mM NALA. T-BCATc mouse colony, *n* = 6 to 9 mice/variant, T-BCATm mouse colony, *n* = 6 mice/variant. In all panels, average ± SEM or ± SD of mixed sex. Statistical significance as determined by a two-tailed Student’s *t*-test: *P < 0.05, **P < 0.01, ***P < 0.001, or ns = no significance.

While deleting BCATc or BCATm mildly impacted *Gata3* and *Rorc* (BCATm only) mRNA expression, the loss of BCATm reduced IL-13 secretion in iT_H_2 cells by 51%, highlighting a specific requirement for mitochondrial transamination (Figure 5A,B). To investigate this further, we induced T_H_2-mediated airway inflammation in T-BCATm^KO^ mice using HDM exposure (Supplementary Figure 2, https://links.lww.com/IN9/A8). Despite the in vitro findings, 4 weeks of exposure did not result in significant differences in lung-infiltrating GATA3^+^ or IL-13^+^ CD44^+^ CD4^+^ effector T cells between T-BCATm^KO^ mice and controls (Supplementary Figure 2, https://links.lww.com/IN9/A8 and Figure 5C). In contrast, NALA-induced leucine antagonism significantly decreased IL-13 (iT_H_2) and IL-17 (iT_H_17) secretion in both control and knockout mice (Figure 5D,E). Overall, we concluded that the BCAT isoenzymes were not required for T_H_2/T_H_17 function. Instead, these results imply that leucine availability may be the key factor driving optimal effector cytokine production.

### 3.5 *Deletion of BCATc causes a transition of Tregs to a T*_*H*_*1-like phenotype*

**Figure 6.**
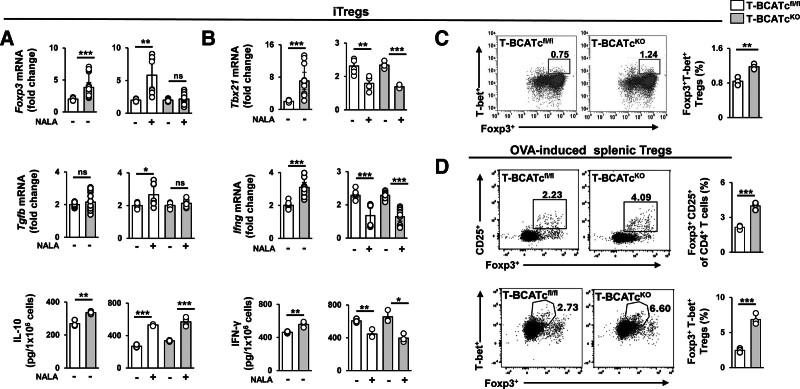
**BCATc deletion promotes a T**_**H**_**1-phenotype in mouse Tregs.** T-BCATc^KO^ and littermate controls were either used as a source of CD4^+^ T cells for in vitro differentiation into iTregs or were challenged with OVA-producing EL4 mouse lymphoma cells for 10 days to induce Treg immune response in vivo. (A) *Foxp3* and *Tgfb*, mRNA expression and secretion of IL-10 from iTregs in the absence or the presence of 10 mM NALA. (B) *Tbx21* and *Ifng* mRNA expression and secretion of IFN-γ from iTreg in the absence or the presence of 10 mM NALA. In A-B, *n* = 10 to 12 mice/variant. (C) Representative flow charts with an average bar graph of CD4^+^ T cells co-expressing Foxp3^+^T-bet^+^ following differentiation to iTregs (*n* = 3 mice/variant). (D) Representative flow charts and average bar graphs showing expression of Foxp3^+^ in CD25^+^CD4^+^ T cells or Tregs co-expressing Foxp3^+^T-bet^+^ following EL4-OVA stimulation in vivo (*n* = 6 mice/variant, a representative experiment is shown). For all panels, average ± SEM or ± SD of mixed sex. Statistical significance as determined by a two-tailed Student’s *t*-test: *P < 0.05, ** *P* < 0.01, *** *P* < 0.001, or ns = no significance.

Investigation on the Treg subset revealed that iTregs from T-BCATc^KO^ mice exhibited significantly higher *Foxp3* mRNA expression (91% increase) and IL-10 secretion (25% increase) compared with iTregs of T-BCATc^fl/fl^ mice (Figure 6A). Furthermore, NALA treatment increased *Foxp3* and *Tgfb* mRNA expressions by 1.9-fold and 34%, and IL-10 release by 98%, in iTregs of T-BCATc^fl/fl^ mice but failed to impact *Foxp3 and Tgfb* mRNA expression in iTregs of T-BCATc^KO^ mice (Figure 6A).

Given the vital role leucine plays in the metabolic reprogramming and activation of T_H_1 cells ^[22,23]^, we next asked if the deletion of BCATc could skew Tregs toward a T_H_1-like phenotype. Indeed, iTregs of T-BCATc^KO^ mice showed significantly enhanced *Tbx21* (2.54-fold increase) and *Ifng* (55% increase) mRNA expression, along with an increased secretion of IFN-γ (21% increase) compared to iTregs of T-BCATc^fl/fl^ mice. Treatment with NALA had the opposite effect (Figure 6B). Consistent with the elevated mRNA levels of *Foxp3 and Tbx21*, iTregs of T-BCATc^KO^ mice co-expressed Foxp3 and T-bet at significantly higher levels than iTregs of T-BCATc^fl/fl^ mice (Figure 6C).

In line with our in vitro findings, OVA stimulation of T-BCATc^KO^ mice led to a significant increase in the splenic Foxp3^+^CD25^+^CD4^+^ T cells (~86% increase) (Figure 6D). Furthermore, T-BCATc^KO^ mice exhibited a significant 1.8-fold increase in splenic Foxp3^+^T-bet^+^ Treg cells compared to cells of T-BCATc^fl/fl^ mice following the OVA challenge (Figure 6D). Collectively, these results demonstrate that the loss of BCATc promotes a transition of Tregs toward a T_H_1-like phenotype. Mechanistically, these effects were independent of mTORC1 pathway activation (data not shown). Instead, the absence of BCATc increased the phosphorylation of the energy sensor AMPK by ~45% (Supplementary Figure 9C, https://links.lww.com/IN9/A8). Furthermore, leucine deprivation of iTregs from T-BCATc^KO^ mice correlated with elevated AMPK protein levels, sustaining higher AMPK activation (Supplementary Figure 9C, https://links.lww.com/IN9/A8). Together, these results suggest that blocking leucine catabolism via BCATc may have an impact on Treg energy metabolism.

### 3.6 Deletion of BCATm mirrors the effect of a loss of BCATc on Tregs

**Figure 7.**
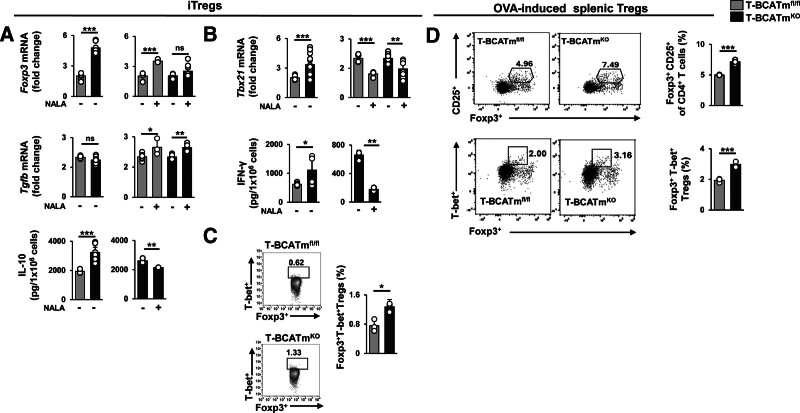
**BCATm deletion causes a similar shift in Tregs toward a T**_**H**_**1-like phenotype as a loss of BCATc.** T-BCATm^KO^ and littermate controls were either used as a source of CD4^+^ T cells for in vitro differentiation into iTregs or were challenged with OVA-producing EL4 mouse lymphoma cells for 10 days to induce Treg immune response in vivo. (A) *Foxp3* and *Tgfb*, mRNA expression and secretion of IL-10 from iTregs in the absence or the presence of 10 mM NALA. (B) *Tbx21* mRNA expression and secretion of IFN-γ from iTregs in the absence or the presence of 10 mM NALA. In A and B, *n* = 3–8 mice/variant. (C) Representative flow charts with an average bar graph of CD4^+^ T cells co-expressing Foxp3^+^T-bet^+^ following differentiation to iTregs (*n* = 3 mice/variant). (D) Representative flow charts and average bar graphs showing expression of Foxp3^+^ in CD25^+^CD4^+^ T cells or Tregs co-expressing Foxp3^+^T-bet^+^ following EL4-OVA stimulation in vivo (*n* = 6 mice/variant, a representative experiment is shown). For all panels, average ± SEM or ± SD of mixed sex. Statistical significance as determined by a two-tailed Student’s *t*-test: *P < 0.05, ** *P* < 0.01, *** *P* < 0.001, or ns = no significance.

Further investigation on the Treg subset revealed that the loss of BCATm from iTregs caused a significant increase in the mRNA expression of *Foxp3* (1.4-fold increase) and *Tbx21* (65% increase) mRNA, and in the release of cytokines IL-10 (66% increase) and IFN-γ (79% increase) (Figure 7A,B). Applying NALA to iTregs increased the mRNA expression of *Foxp3* (in T-BCATm^fl/fl^ mice only) and *Tgfb*, but reduced the expression of *Tbx21*, or the release of IL-10 or IFN-γ, as compared with untreated cells (Figure 7A,B). Except for the NALA effects on IL-10, these results were reminiscent of the findings obtained for the T-BCATc^KO^ mouse (refer to Figure 6A,B). Moreover, we detected a higher percentage of Foxp3^+^T-bet^+^ Tregs (~67% increase) in T-BCATm^KO^ mice compared to T-BCATm^fl/fl^ mice following in vitro stimulation (Figure 7C). Lastly, OVA-induction caused a significant increase by 43% of Foxp3^+^CD25^+^CD4^+^ T cells and a higher percentage of Tregs co-expressing Foxp3 and T-bet (54% increase) in spleens of T-BCATm^KO^ mice (Figure 7D).

Mechanistically, BCATm-deficient iTregs exhibited a 95% increase in AMPK activation (Supplementary Figure 9D, https://links.lww.com/IN9/A8), while mTORC1 signaling remained unaffected (data not shown). Taken together, the results suggest that a single loss of either BCAT isoenzyme upregulates the energy sensor AMPK and is sufficient to stimulate a shift of Tregs to T_H_1-like phenotype.

### 3.7 Human BCAT1 and BCAT2 correlate with distinct combinations of KEGG pathways and associated genes in T helper subsets

**Figure 8.**
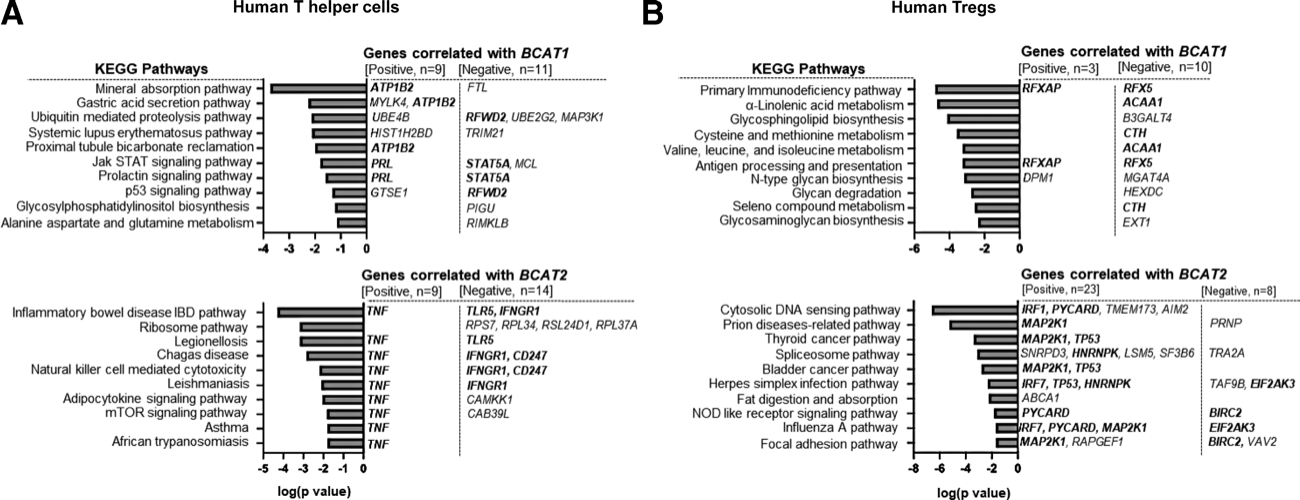
**KEGG pathway analysis of genes correlated with *BCAT1* or *BCAT2* in human tonsils.** Positive and negative gene correlations based on KEGG pathway analysis of (A) activated T helper cells and (B) Tregs. Genes that significantly correlated with *BCAT1* or *BCAT2*, but appeared associated with more than one KEGG pathway, are presented in bold. *n* = 5 specimens, correlation *P* value cutoff <0.01.

To determine if the human orthologs of *Bcat1* and *Bcat2* map to distinct pathways as suggested by their differential impact on mouse T helper subsets, we analyzed public gene expression profiles of human CD4^+^ T cell subsets. KEGG pathway analysis with human *BCAT1* and *BCAT2* as the reporter genes revealed that these genes correlated with distinct combinations of pathways and associated genes in activated T helper and Tregs (Figure 8, Supplementary Table 3, https://links.lww.com/IN9/A9). Analysis of the top 10 KEGG pathways in activated T helper cells revealed 20 and 23 gene correlations with *BCAT1* and *BCAT2*, respectively. Among those, *BCAT1* appeared significantly correlated with genes involved in processes, such as mineral absorption, ubiquitin-mediated proteolysis, and the Jak–STAT pathway, while *BCAT2* associated with genes involved in the inflammatory bowel disease pathway, as well as the ribosome and mTOR signaling pathway (Figure 8A). Supplementary Table 3, https://links.lww.com/IN9/A9, details these genes along with their respective correlation coefficients (*R*) and *P* values.

Similarly, the KEGG pathway analysis revealed that *BCAT1* and *BCAT2* correlated with genes involved in distinct and nonoverlapping pathways in human Tregs (Figure 8B). The ten most prominent KEGG pathways and associated genes indicated that *BCAT1* was mostly negatively associated (*n* = 10 negative correlations), while *BCAT2* was mostly positively associated (*n* = 23 positive correlations) with genes from the analyzed pathways (Figure 8B). Full gene descriptions, including *R* and *P* values, are provided in Supplementary Table 4, https://links.lww.com/IN9/A9. Taken together, these data further suggest that *BCAT1* and *BCAT2* may have noncanonical roles in T cells.

## 4. Discussion

Nutrient utilization is of great importance for proper T-cell function across tissue microenvironments and disease states. CD4^+^ T-cell subsets commit to their lineages using diverse metabolic strategies, where T_H_1, T_H_2, and T_H_17 cells upregulate glycolytic metabolism. In contrast, Tregs rely on oxidative phosphorylation and fatty acid oxidation to maintain suppressive functions ^[24,25]^. Utilization of amino acids varies considerably across T-cell subsets. Our understanding of amino acids extends beyond their roles as the building blocks of proteins because they can influence the T-cell metabolic reprogramming and T-cell fate ^[26,27]^.

It is well known that arginine is important for T_H_1 and T_H_17 differentiation, while low tryptophan levels promote the development of Tregs ^[28,29]^. Another well-established immunomodulatory amino acid, glutamine, is important for T_H_1 and T_H_17 cells, but to a lesser extent for Tregs ^[30]^. In contrast, BCAAs have received scarce scientific attention as to their role in T-cell subset differentiation, undermining their importance for T-cell metabolic fitness and effector function. In 2009, Zheng et al illustrated that leucine availability was closely linked to T cell functionality. In this study, blockade of leucine-mediated mTORC1 activation via NALA competitive antagonism impaired T-cell metabolic machinery in response to signal 1 + 2, leading to an anergic state with impaired function. At concentrations of 10 mM or higher, NALA significantly suppressed the ability of T_H_1 cells to release IL-2 or IFN-γ, an effect that was comparable to that of the mTORC1 inhibitor, rapamycin ^[22]^. Similarly, 20 mM NALA mimicked a treatment with 200 nM rapamycin or leucine starvation in inhibiting mTORC1 signaling and cell proliferation in human Jurkat T cells ^[14]^. In this study, NALA induced G1 phase cell-cycle arrest by suppressing the degradation of the inhibitor p27 and reducing the phosphorylation of the retinoblastoma protein (Rb). The effects, mediated via the leucine–mTORC1 axis, overlapped with those of rapamycin ^[14]^. Similar findings in mouse EL4 T-cell lymphoma demonstrated that NALA and leucine-low medium have comparable effects on mTORC1, glycolytic capacity, and cell growth ^[31]^. However, such results should be interpreted with caution until the specific receptor or transporter mediating NALA’s effects is identified.

The heterodimeric complex composed of Slc7a5 (LAT1) and its chaperone Slc3a2 (CD98hc) is the primary transporter system mediating BCAA uptake in activated T-cell subsets ^[5,6]^. Sinclair et al demonstrated that mouse CD4^+^ T cells, deficient in the leucine transporter, Slc7a5, and treated with T_H_1 or T_H_17 skewing cytokines, failed to generate T_H_1 or T_H_17 cells but were able to generate Foxp3^+^ Tregs upon addition of TGF-β. Moreover, these authors’ findings pointed toward a more significant role of leucine than previously thought, as the failed leucine uptake in Slc7a5-null T cells led to a metabolic catastrophe and prevented clonal expansion. This was driven by the inability of Slc7a5-null T cells to express the c-Myc protein and to upregulate glucose and glutamine metabolism for T-cell proliferation and differentiation ^[5]^. Applying JPH203 to inhibit leucine uptake in human T cells further demonstrated a marked reduction in the release of IFN-γ, IL-4, and IL-17, signifying the importance of leucine uptake in human T cells ^[32]^. In contrast, a study by Ikeda et al has shown that the Slc3a2 transporter is required for BCAA-dependent maintenance of Tregs. The study demonstrated that blocking isoleucine uptake through Slc3a2 resulted in defective replication and decreased number of Tregs expressing Foxp3, signifying the importance of isoleucine for Treg maintenance ^[6]^.

Here, we offered a novel discovery addressing a missing link between BCAA catabolism and CD4^+^ T-cell subset differentiation by using our newly generated T-cell conditional knockout mouse models of BCATc and its mitochondrial isoenzyme BCATm. Because BCATc is an inducible enzyme upon T-cell activation ^[4]^, we did not anticipate an impact of a loss of BCATc on inflammatory markers or CD4^+^ T-cell populations in the naïve animal. However, it was somewhat surprising to discover that a loss of BCATm expression did not produce a significant phenotype in CD4^+^ T cells of the naïve animal. While BCATm is constitutively expressed and contributes to the basic metabolic needs of naïve T cells ^[7]^, this isoenzyme did not appear indispensable.

Before assessing how the loss of each BCAT isoenzyme impacts CD4^+^ T-cell subsets, we activated WT mouse CD4^+^ T cells using lineage-skewing cytokines. We found that BCATc expression decreased in all CD4^+^ T-cell subsets relative to T_H_0 cells. Conversely, BCATm expression was context-dependent, showing downregulation in T_H_17 and Tregs, but upregulation in T_H_1 and T_H_2 cells—indicating specialized mitochondrial BCAA catabolism requirements. In fact, when BCATm was deleted from T_H_2 cells, they released significantly less IL-13, revealing a potential functional requirement of T_H_2 cells for BCATm. However, these in vitro findings were not reproduced in vivo using an HDM-induced model of lung inflammation, suggesting that BCATm is dispensable for T_H_2 lineage commitment.

A major finding of this study was the impact of a loss of BCATc, but not that of BCATm, on T_H_1 differentiation and function. BCATc-deficient T_H_1 cells demonstrated increased expression of T-bet and enhanced functionality as evidenced by significantly increased IFN-γ secretion. Applying NALA had a suppressive effect on IFN-γ released by T_H_1 cells regardless of genotype, a finding consistent with earlier reports on T_H_1 cells and their requirement for leucine ^[4,22]^. Partly, the role of leucine in T_H_1 subset differentiation could be explained by its promoting effects toward mTORC1. Previously, we have shown that activated T_H_1 cells had improved glycolytic rate and glycolytic capacity due to upregulation of mTORC1 signaling in the absence of BCATc. According to this report, a loss of BCATc expression led to increased intracellular concentrations of leucine, causing activation of the ribosomal S6 protein, which serves as a downstream target of mTORC1 pathway ^[4]^. These effects were reversed by NALA and agreed with other studies on the leucine-dependent upregulation of mTORC1 in T cells ^[5,22]^. Multiple reports, focused on genetic perturbations in key components constituting mTORC1 and mTORC2 signaling cascades, have shown the impact of mTOR signaling on T_H_ lineage commitment. CD4^+^ T cells, deficient in *Frap1* (encodes mTOR), failed to differentiate into T_H_1, T_H_2, and T_H_17 cells, yet they differentiated to Tregs ^[33]^. CD4^+^ T cells, deficient in RICTOR (inhibiting mTORC2 activity), failed to generate T_H_2 cells, while retaining T_H_1 and T_H_17 differentiation ^[34]^. Further, CD4^+^ T cells deficient in Rheb failed to differentiate into T_H_1, T_H_17, or Tregs ^[33]^. Oppositely, a deficiency in TSC1 or TSC2 increased the potential to differentiate to T_H_1 and T_H_17 cells, while facilitating a shift from Tregs to T_H_17 cells ^[35]^. Such reports identified paradigms within T cell metabolic reprogramming, which are unique for each subset ^[36]^. Our current report complements these studies, as evidenced by the upregulation of the mTORC1 pathway in T_H_1 cells deficient of BCATc. Notably, this upregulation was differentially affected by leucine deprivation: 4EBP-1 phosphorylation was downregulated, while S6 phosphorylation remained unaffected in T_H_1 cells of T-BCATc^KO^ mice. These results suggest a noncanonical and potentially noncatalytic role for BCATc in regulating T_H_1 cell signaling. Conversely, BCATm differentially affected the S6 and 4EBP-1 branches of the mTORC1 pathway in T_H_1 cells. Because these branches appeared disproportionately affected, a deficiency in BCATm may ultimately be insufficient to affect T_H_1 cells and their functional capacity. These findings are supported by a recent study identifying BCATm as a key mitochondrial gene for the metabolic reprogramming of activated CD4^+^ T cells. While losing BCATm increased mitochondrial respiration, it uncoupled oxygen consumption from ATP synthesis. This paradigm forced the cells to adopt a highly glycolytic phenotype to help prevent exhaustion and maintain functionality via the AMPK-mTOR axis ^[37]^.

Our investigation also showed that leucine antagonism with NALA impaired T_H_2 and T_H_17 functionality. Notably, while our study was the first to connect BCAAs and T_H_2 regulation, Kang et al illustrated a potential role of modulating leucine uptake or leucine metabolism in human T_H_17 cells. In this study, co-stimulated CD4^+^ memory T cells, treated with Bi2, an inhibitor of BCATc, reduced IL-17 secretion. In addition, the same authors showed that removal of all BCAAs from the growth medium reduced, while alternatively applying increasing concentrations of leucine increased the secretion of IL-17 from CD4^+^ memory T cells. A lack of response of iT_H_17 cells to a loss of BCATc in our study may likely be explained by the differences that may arise from the unique metabolic requirements for differentiation of naïve CD4^+^ T cells to a T_H_17 phenotype vs the co-stimulation of CD4^+^ memory T cells ^[38]^.

A second major finding of this study was that deletion of either BCAT isoenzyme caused Tregs to skew toward a T_H_1-like phenotype. This was evident by our in vitro and in vivo models, which highlighted that removing BCATc or BCATm from Tregs increased *Tbx21* mRNA, enhanced IFN-γ secretion, and promoted a Foxp3^+^T-bet^+^ Treg phenotype. Importantly, pharmacological inhibition of leucine uptake by NALA reversed the effects on *Tbx21* mRNA and IFN-γ in Tregs deficient of BCATc or BCATm, suggesting the observed changes in Treg differentiation and function were dependent on leucine availability. Interestingly, the addition of NALA caused significant increases in *Foxp3* and *Tgfb* mRNA levels and significant release of IL-10 from Tregs expressing normal levels of BCATc and BCATm, but failed to induce similar changes in *Foxp3* mRNA in Tregs deficient in BCATc or BCATm. This implies that the leucine-dependent impact on Treg ability to express Foxp3 was interrupted by the single loss of a BCAT isoenzyme. Past reports have shown that Tregs acquire an effector T_H_1-like phenotype under a diverse set of inflammatory conditions ^[39]^. Commonly acquired ability of these cells is the capacity to produce IFN-γ and lose suppressive activity while maintaining Foxp3 expression ^[40]^. In vitro stimulation in the presence of IL-12 of Tregs from healthy individuals or virial infections/autoimmune conditions stimulates the generation of T_H_1-Tregs ^[39,41]^. The current report is in alignment with these studies showing that Foxp3 expression was maintained upon the loss of either BCAT or when leucine antagonism via NALA was applied. Similar to leucine antagonism, glutamine restriction leads to high expression of Foxp3 in activated human CD4^+^ T cells. In contrast, blocking glutamine synthetase, the enzyme that catalyzes the conversion of glutamate into glutamine, can abolish this effect ^[42]^. While deficiency of leucine or glutamine transporters has no effects on naïve T-cell differentiation into Tregs ^[43,44]^, the metabolic reprogramming of Tregs appears dependent upon amino acid-induced mTORC1 signaling ^[45]^. Shi and coauthors showed that Rag A/B GTPases are central regulators of leucine and arginine-dependent mTORC1 activation of effector Tregs. In this study, acute depletion of leucine and arginine, and to a lesser extent, glutamine, led to impaired mTORC1 signaling in Tregs. However, the effects of leucine and arginine to license and sustain Tregs fitness were dependent on functional RagA and RagB proteins. Mice lacking these factors showed reduced frequency and number of Foxp3^+^ Tregs and developed a Scurfy-like autoimmune disease. Applying a leucine-deficient diet, however, did not affect the number of effector Foxp3^+^ Tregs in spleens or lymph nodes of WT mice ^[45]^. In the current report, we did not find changes in mTORC1 signaling in Treg-deficient of BCATc or BCATm. These results imply that mTORC1 pathway does not take part in the effects of leucine degradation on Tregs. In opposite, the loss of either BCAT contributed to a significant increase in the activity of the energy sensor AMPK. Furthermore, exposure of BCATc-deficient Tregs to leucine-free medium correlated with elevated concentration and activity of AMPK. This is consistent with previous reports on the demand of both immune and nonimmune cells on active BCAA metabolism for energy and TCA cycle intermediates ^[37,46–48]^. In Tregs, specifically, the role of AMPK has been described as necessary for the adaptation of these cells to micromovements that induce stress, such as malignancy or viral infections ^[49]^. Mechanistically, AMPK function in Tregs includes epigenetic and metabolic changes that support the immunosuppressive function of Tregs ^[49]^. Our findings provide initial clues on the role of BCAA metabolism in this process but need further investigation.

**Figure 9.**
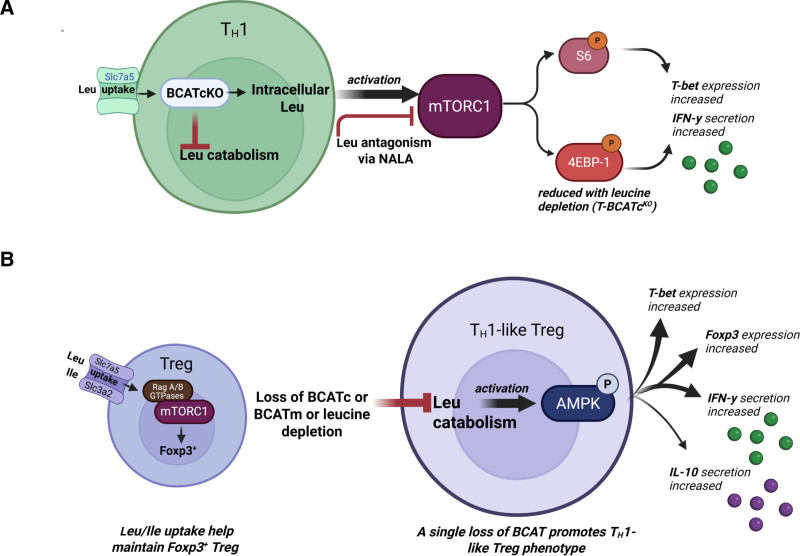
**Simplified model of BCAA catabolism in T-cell differentiation and function.** (A) In T_H_1 cells, loss of BCATc promotes differentiation and effector function in an mTORC1-dependent manner. BCATc-deficient T_H_1 cells exhibit elevated intracellular leucine, which upregulates mTORC1 signaling (evidenced by increased phosphorylation of S6 and 4EBP-1), subsequently increasing T-bet expression and IFN-γ secretion. Leucine antagonism via NALA exerts opposing effects regardless of BCATc expression. However, leucine depletion does not affect S6 phosphorylation in BCATc-deficient T_H_1 cells of the T-BCATc^KO^ mice, suggesting a non-canonical signaling role for BCATc. (B) In Tregs, leucine or isoleucine uptake maintains the Foxp3^+^ phenotype. The role of leucine is dependent on the activation of RagA/B GTPases to upregulate mTORC1. While the loss of BCATc or BCATm does not alter mTORC1 activity in Tregs, it upregulates the energy sensor AMPK. This metabolic shift correlates with the adoption of a T_H_1-like Treg phenotype, characterized by elevated T-bet and Foxp3 expression alongside increased secretion of IFN-γ and IL-10. This schematic is based on findings reported in this study and as reported previously^4,5,6,45^. Created with BioRender. BCAA, branched-chain amino acid; BCATc, cytosolic branched-chain aminotransferase; BCATm, mitochondrial branched-chain aminotransferase; IFN, interferon; IL, interleukin; mTORC1, mammalian target of rapamycin complex 1; NALA, *N*-acetyl leucine amide.

In summary, this research served to establish the foundational role of BCATc in CD4^+^ T-cell differentiation and function while comparing with its constitutively expressed isoenzyme, BCATm. According to our investigation, BCATc is the dominant isoenzyme in T-cell differentiation toward the T_H_1 subset. The findings direct toward mTORC1-dependant immunoregulatory role of BCATc on the ability of T_H_1 cells to commit to their lineage and maintain functional integrity (Figure 9). In contrast, a single loss of either BCAT isoenzyme appears sufficient to skew Tregs to a T_H_1-like phenotype. These effects may involve energy adjustment via AMPK to allow Tregs to maintain Foxp3 while expressing higher levels of T-bet and IFN-γ and is highlighted in the simplified model in Figure 9. While future research is needed to uncover the exact mechanism of this switch, our transcriptomic analysis suggests distinct, noncanonical functions for each BCAT isoenzyme within the Treg subset.

Although, this manuscript provides a comprehensive investigation into leucine catabolism and antagonism during CD4^+^ T cell lineage commitment, further research is necessary to: (1) fully characterize the functional state and suppressive activity of BCATc- and BCATm-deficient T_H_1-like Tregs, (2) account for any biological effects of NALA that may occur independently of leucine antagonism, (3) investigate noncanonical or noncatalytic roles of BCATc/BCATm, such as their impact on redox balance or alternative signaling pathways, (4) experimentally validate the human transcriptomic analysis of T helper and Tregs, which currently remains exploratory.

## 5. Conclusions

Disrupting the first step of leucine degradation impacts the metabolic reprogramming and, consequently, the differentiation of T cells. Because the loss of BCATc significantly modulates T_H_1 cells, and a loss of either BCATc or BCATm impacts Tregs, these enzymes represent promising targets for modulating T-cell-driven responses across various diseases. Our team is currently investigating the BCATc-defined signature of T-cell subsets within the tumor microenvironment of T-BCATc^KO^ mice challenged with lymphoma and treated with monoclonal therapies. These studies aim to clarify the therapeutic potential of targeting BCATc to enhance antitumoral immunity.

## Author contributions

T.J.W. and E.A.A. designed, directed, as well as performed most of the experiments, analyzed the data, and wrote the manuscript. C.M.A. conducted the RT-PCR analysis to validate the genotypes, followed by phenotyping, of the transgenic mouse colonies. T.Z.K. conducted experiments with WT mice subjected to T cell subset skewing cytokines. A.H.D. and M.A.M, in collaboration with T.J.W., conducted the flow analysis of the T-BCATc^KO^ and T-BCATm^KO^ mouse colonies. T.J.W. and E.A.A., along with T.J.K, A.E.K., and M.E.F., conducted the OVA-induced in vivo stimulation of the T-BCATc^KO^ and T-BCATm^KO^ mouse colonies. B.H.L. and J.P.S. performed the dust-mite experiment with the T-BCATm^KO^ mouse colony. All authors participated in data interpretation, reviewed, and approved the manuscript.

## Conflicts of interest

The authors declare no conflict of interest.

## Funding

This work was supported through NIH NCI 1R15CA249796-01A1 awarded to E.A.A. and Des Moines University; DMU 145-3610 [The Elsie Lee Cancer Research Fund], IOER 09-16-06 and IOER 03-18-02 awarded to E.A.A.

## Acknowledgments

The authors thank Mike Boyer for genotyping the mouse colonies and assisting with reagents preparation, as well as Rebecah Betar, DO’23, and Alex Martin, DO’23, for assisting with some of the western blotting and organ collection analysis during the mouse colonies validation stage.

## Supplementary Material
