# Supplementary material for: A loss of the cytosolic branched-chain aminotransferase, BCATc, enhances Th1 differentiation and skews Tregs to acquire a Th1-like phenotype
Source: Immunometabolism (Cobham). 2026 Jul 13;8(3):e00084. doi: 10.1097/IN9.0000000000000084 (PMC13361958; doi:10.1097/IN9.0000000000000084)
Supplement: Supplementary file 1 [file in9-8-e00084-s001.pdf]

Supplementary Table 1. PCR primer sequences

Gene	Forward	Reverse
**Genotyping PCR primer sequences**		
**Gene**	**Forward**	**Reverse**
Bcat1	5’ GTCTGTGGAGGTCTCAGGTCAGCTTG 3’	5’ ATCCCAGAAGGTCACCCAAACAAA 3’
Bcat2	5’ GTTCTCAAGGTGGTGGGTGT 3’	5’ TCCCTGGTGCCTGACACTAAA 3’
	-	5’ AGAAGCCACAGGGGAAATGT 3’
CD4Cre	5’ GTTCTTTGTATATATTGAATGTTAGCC 3’	-
WT-CD4Cre	-	5’ TATGCTCTAAGGACAAGAATTGACA 3’
Mut-CD4Cre	-	5’ CTTTGCAGAGGGCTAACAGC 3’
**Inflammatory marker qRT-PCR primer sequences**		
**Gene**	**Forward**	**Reverse**
Ccl8	5’ GGGTGCTGAAAAGCTACGAG 3’	5’ GAGAGACATACCCTGCTTGG 3’
Slpi	5’ GCGTCAAAACTCAGGCAAGA 3’	5’ AGATCGGTGAATGCTGAGCCA 3’
Tnfa	*5’* TCTCATCCCATGCCTAACTGC *3’*	*5’* CGTGGGTTGGACAGATGAATG *3’*
Ifng	*5’* CAGCAACAGCAAGGCGAAA *3’*	*5’* CTGGACCTGTGGGTTGTTGAC *3’*
**$CD4^+$ T cell lineage specific marker qRT-PCR primer sequences**		
**Gene**	**Forward**	**Reverse**
Foxp3	5’ TTCGAGGAGCCAGAAGAGTTTC 3’	5’ GGGCCTTGCCTTTCTCATC 3’
Tbx21	5’ GCCAGGGAACCGCTTATATG 3’	5’ GACGATCATCTGGGTCACATTGT 3’
Tgfb1	5’ GCTCTTGTGACAGCAAAGATAACAA 3’	5’ CGCCCCGACGTTTGG 3’
Gata3	5’ GCCTGCGGACTCTACCATAA 3’	5’ AGGATGTCCCTGCTCTCCTT 3’
Rorc	5’ CAAGTCATCTGGGATCCACTAC 3’	5’ TGCAGGAGTAGGCCACATTACA 3’
Bcat1	5’ GTACATCAGAGCCTGGAAAGG 3’	5’ ACCTGCTGACAGCCATTCTC 3’
Bcat2	5’ TGGTCTGCACTACTCTCTGC 3’	5’ TTGTCAAAGTCTGGCAGGCA 3’
Ef1a	5’ GACAGCAAAAACGACCCACC 3’	5’ ATCCAGAACAGGAGCGTAGC 3’

Supplementary Table 2.

Body and selected organ weights of T-BCATc$^{fl/fl}$ and T-BCATc^{KO} mice

	**fl/fl**		**KO**	
Measurement (g)	Male	Female	Male	Female
**Body weight**	25.9 ± 0.62	20.8 ± 0.29	26.7 ± 0.43	21.2 ± 0.40
**Spleen**	0.075 ± 0.00	0.104 ± 0.00	0.069 ± 0.00	0.098 ± 0.00
**Thymus**	0.042 ± 0.00	0.059 ± 0.00	0.035 ± 0.00	0.058 ± 0.01
**Lungs**	0.156 ± 0.01	0.202 ± 0.01	0.162 ± 0.02	0.189 ± 0.01
**Kidneys**	0.319 ± 0.01	0.303 ± 0.01	0.300 ± 0.02	0.279 ± 0.02
**Heart**	0.144 ± 0.01	0.157 ± 0.00	0.132 ± 0.01	0.152 ± 0.02
**Brain**	0.394 ± 0.01	0.509 ± 0.02	0.349 ± 0.05	0.529 ± 0.04

Body and selected organ weights of T-BCATm$^{fl/fl}$ and T-BCATm^{KO} mice

	**fl/fl**		**KO**	
**Body weight**	29.2 ± 0.40	23.5 ± 0.27	29.4 ± 0.65	23.8 ± 0.39
**Spleen**	0.068 ± 0.00	0.106 ± 0.00	0.061 ± 0.00*	0.098 ± 0.00
**Thymus**	0.038 ± 0.00	0.045 ± 0.00	0.034 ± 0.00	0.038 ± 0.00
**Lungs**	0.178 ± 0.02	0.204 ± 0.01	0.156 ± 0.01	0.200 ± 0.01
**Kidneys**	0.343 ± 0.02	0.312 ± 0.02	0.292 ± 0.01	0.271 ± 0.01
**Heart**	0.140 ± 0.01	0.159 ± 0.01	0.132 ± 0.01	0.143 ± 0.01
**Brain**	0.376 ± 0.01	0.490 ± 0.02	0.374 ± 0.01	0.470 ± 0.03

Body and organ weights of naive T-BCATc$^{fl/fl}$, T-BCATc^{KO}, T-BCATm$^{fl/fl}$ and T-BCATm^{KO} male and female mice. Averaged age 14±3 weeks, n≥7 mice/sex/group. Data represent mean ± SEM. Statistical significance as determined by a two-tailed Student's t-test: *$P < 0.05$ as compared with T-BCATm $^{fl/fl}$ male mice. The organ weights were normalized to 25 g body weight.

Supplementary Table 3. KEGG pathway analysis of activated Th cells from human tonsils (n=5)

Pathways and genes correlated with *BCAT1* (human gene for BCATc)

	Gene	*Gene description*	*R*-value	*P*-value
Mineral absorption	***ATP1B2***	*ATPase, Na^{+}/K^{+} transporting, β2*	0.962	0.00867
	FTL	*Ferritin, light polypeptide*	-0.971	0.00597
Gastric acid secretion	*MYLK4*	*Myosin light chain kinase 4*	0.960	0.00942
	ATP1B2	*ATPase, Na^{+}/K^{+} transporting, β2*	0.962	0.00867
Ubiquitin mediated proteolysis	*UBE4B*	*Ubiquitination factor E4B*	0.980	0.00345
	RFWD2	*Ring finger and WD2, E3 ubiquitin protein ligase*	-0.988	0.00163
	UBE2G2	*Ubiquitin-conjugating enzyme E2G 2*	-0.970	0.00629
	MAP3K1	*Mitogen-activated protein kinase 1*	-0.963	0.00865
Systemic lupus erythematosus	*HIST1H2BD*	*Histone cluster 1, H2bd*	0.976	0.00448
	TRIM21	*Tripartite motif containing 21*	-0.964	0.00819
Proximal tubule bicarbonate reclamation	***ATP1B2***	*ATPase, Na+/K+ transporting, β2*	0.962	0.00867
Jak STAT signaling	***PRL***	*Prolactin*	0.970	0.00625
	STAT5A	*Signal transducer and activator of transcription 5A*	-0.993	0.00066
	MCL1	*Myeloid cell leukemia 1*	-0.985	0.00212
Prolactin signaling	***PRL***	*Prolactin*	0.970	0.00625
	STAT5A	*Signal transducer and activator of transcription 5A*	-0.993	0.00066
p53 signaling	*GTSE1*	*G-2 and S-phase expressed 1*	0.996	0.00750
	RFWD2	*Ring finger and WD2, E3 ubiquitin protein ligase*	-0.988	0.00163
Glycosylphosphatidylinositol biosynthesis	*PIGU*	*Phosphatidylinositol glycan anchor, class U*	-0.985	0.00224
Alanine aspartate and glutamate metabolism	*RIMKLB*	*Ribosomal modification protein rimK-member B*	-0.967	0.00700

Pathways and genes correlated with *BCAT2* (human gene for BCATm)

	Gene	*Gene description*	*R*-value	*P*-value
Inflammatory bowel disease IBD	***TNF***	*Tumor necrosis factor*	0.987	0.00172
	TLR5	*Toll-like receptor 5*	-0.994	0.00060
	IFNGR1	*Interferon gamma receptor1*	-0.975	0.00485
Ribosome pathway	*RPS7*	*Ribosomal protein S7*	-0.994	0.00057
	RPL34	*Ribosomal protein L34*	-0.970	0.00632
	RSL24D1	*Ribosomal L24 domain containing 1*	-0.969	0.00642
	RPL37A	*Ribosomal protein L37a*	-0.961	0.00922
Legionellosis	***TNF***	*Tumor necrosis factor*	0.987	0.00172
	TLR5	*Toll-like receptor 5*	-0.994	0.00060
Chagas disease	***TNF***	*Tumor necrosis factor*	0.987	0.00172
	IFNGR1	*Interferon gamma receptor1*	-0.975	0.00485
	CD247	*CD247 molecule*	-0.971	0.00586
Natural killer cell mediated cytotoxicity	***TNF***	*Tumor necrosis factor*	0.987	0.00172
	IFNGR1	*Interferon gamma receptor 1*	-0.975	0.00485
	CD247	*CD247 molecule*	-0.971	0.00586
Leishmaniasis	***TNF***	*Tumor necrosis factor*	0.987	0.00172
	IFNGR1	*Interferon gamma receptor 1*	-0.975	0.00485
Adipocytokine signaling	***TNF***	*Tumor necrosis factor*	0.987	0.00172
	CAMKK1	*Calcium/calmodulin-dependent protein kinase 1*	-0.989	0.00133
mTOR signaling	***TNF***	*Tumor necrosis factor*	0.987	0.00172
	CAB39L	*Calcium binding protein 39-like*	-0.973	0.00517
Asthma	***TNF***	*Tumor necrosis factor*	0.987	0.00172
African trypanosomiasis	***TNF***	*Tumor necrosis factor*	0.987	0.00172

Source, R2: Genomics Analysis and Visualization Platform (http://r2.amc.nl). Genes that significantly correlated with *BCAT1* or *BCAT2*, but appeared associated with more than one KEGG pathway, are presented in bold.

Supplementary Table 4. KEGG pathway analysis of Tregs from human tonsils (n=5)

Pathways and genes correlated with *BCAT1*

	Gene	Gene description	*R*-value	*P*-value
Primary immunodeficiency	***RFXAP***	*Regulatory factor X-associated protein*	0.974	0.00492
	RFX5	*Regulatory factor X5*	-0.972	0.00566
α-Linolenic acid metabolism	***ACAA1***	*Acetyl-CoA acyltransferase 1*	-0.962	0.00870
Glycosphingolipid biosynthesis	*B3GALT4*	*β-1,3-galactosyltransferase 4*	-0.981	0.00310
Cysteine and methionine metabolism	***CTH***	*Cystathionine γ-lyase*	-0.977	0.00410
Valine, leucine, isoleucine degradation	***ACAA1***	*Acetyl-CoA acyltransferase 1*	-0.962	0.00870
Antigen processing and presentation	***RFXAP***	*Regulatory factor X-associated protein*	0.974	0.00492
	RFX5	*Regulatory factor X5*	-0.972	0.00566
N-type glycan biosynthesis	*DPM1*	*Dolichyl-phosphate mannosyltransferase 1*	0.973	0.00530
	MGAT4A	*Mannosyl-glycoprotein 4-β-N acetylglucosaminyltransferase A*	-0.992	0.00079
Glycan degradation	*HEXDC*	*Hexosaminidase (catalytic domain)*	-0.983	0.00271
Seleno compound metabolism	***CTH***	*Cystathionine γ-lyase*	-0.977	0.00410
Glycosaminoglycan biosynthesis	*EXT1*	*Exostosin glycosyltransferase 1*	-0.977	0.00420

Pathways and genes correlated with *BCAT2*

	Gene	Gene description	*R*-value	*P*-value
Cytosolic DNA sensing	***IRF7***	*Interferon regulatory factor 7.*	0.997	0.000229
	PYCARD	*Adaptor protein with PYD and CARD domains*	0.994	0.000524
	TMEM173	*Transmembrane protein 173*	0.988	0.001550
	AIM2	*Absent in melanoma 2*	0.959	0.009900
Prion diseases-related	***MAP2K1***	*Mitogen-activated protein kinase 1*	0.991	0.001050
	PRNP	*Prion protein*	-0.960	0.009480
Thyroid cancer pathway	***MAP2K1***	*Mitogen-activated protein kinase 1*	0.991	0.001050
	TP53	*Tumor protein p53*	0.991	0.001080
Spliceosome pathway	*SNRPD3*	*Small nuclear ribonucleoprotein D3*	0.996	0.000278
	HNRNPK	*Heterogeneous nuclear ribonucleoprotein K*	0.983	0.002760
	LSM5	*U6 small nuclear RNA associated*	0.975	0.004800
	SF3B6	*Splicing factor 3B, subunit 6,*	0.967	0.007050
	TRA2A	*Transformer 2α*	-0.999	0.000026
Bladder cancer pathway	***MAP2K1***	*Mitogen-activated protein kinase 1*	0.991	0.001050
	TP53	*Tumor protein p53*	0.991	0.001080
Herpes simplex infection	***IRF7***	*Interferon regulatory factor 7*	0.997	0.000229
	TP53	*Tumor protein p53*	0.991	0.001080
	HNRNPK	*Heterogeneous nuclear ribonucleoprotein K*	0.983	0.002760
	TAF9B	*TBP-associated factor, 9B*	-0.993	0.000738
	EIF2AK3	*Eukaryotic translation initiation factor 2α kinase 3*	-0.971	0.006050
Fat digestion and absorption	*ABCA1*	*ATP-binding cassette, sub-family A*	0.974	0.005070
NOD like receptor signaling	***PYCARD***	*Adaptor protein with PYD and CARD domains*	0.994	0.000524
	BIRC2	*Baculoviral IAP repeat containing 2*	-0.974	0.004960
Influenza A pathway	***IRF7***	*Interferon regulatory factor 7*	0.997	0.000229
	PYCARD	*Adaptor protein with PYD and CARD domains*	0.994	0.000524
	MAP2K1	*Mitogen-activated protein kinase 1*	0.991	0.001050
	EIF2AK3	*Eukaryotic translation initiation factor 2α kinase 3*	-0.971	0.006050
Focal adhesion	***MAP2K1***	*Mitogen-activated protein kinase 1*	0.991	0.001050
	RAPGEF1	*Rap guanine nucleotide exchange factor 1*	0.965	0.007940
	BIRC2	*Baculoviral IAP repeat containing 2*	-0.974	0.004960
	VAV2	*Guanine nucleotide exchange factor*	-0.972	0.005620

Source, R2: Genomics Analysis and Visualization Platform (http://r2.amc.nl). Genes that significantly correlated with *BCAT1* or *BCAT2*, but appeared associated with more than one KEGG pathway, are presented in bold.
