# Supplementary material for: A loss of the cytosolic branched-chain aminotransferase, BCATc, enhances Th1 differentiation and skews Tregs to acquire a Th1-like phenotype
Source: Immunometabolism (Cobham). 2026 Jul 13;8(3):e00084. doi: 10.1097/IN9.0000000000000084 (PMC13361958; doi:10.1097/IN9.0000000000000084)
Supplement: Supplementary file 2 [file in9-8-e00084-s002.pdf]

Supplementary Fig. 1

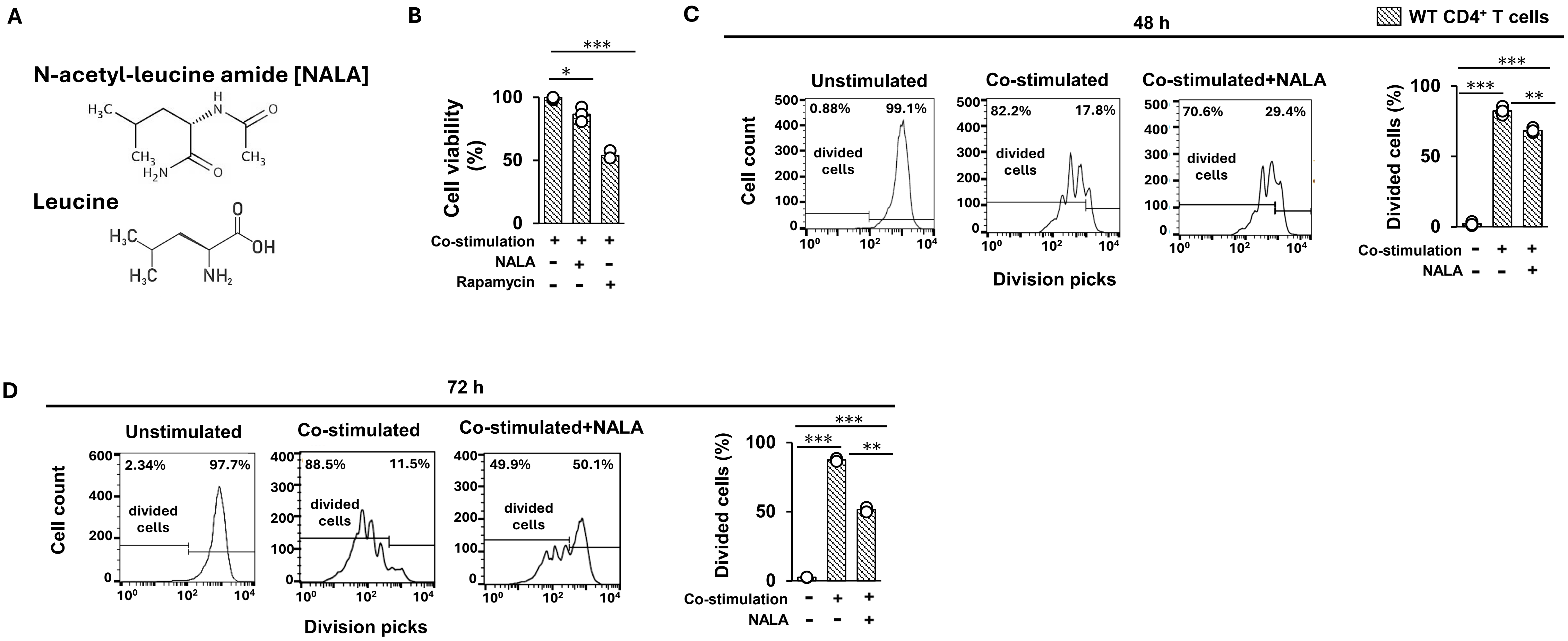

Supplementary Figure 1. Characterization of CD4⁺ T cell viability and proliferation in response to NALA exposure. In **A**, the chemical structure of NALA as compared to that of leucine. In **B-D**, WT CD4⁺ T cells, isolated from mouse spleens and lymph nodes, were left unstimulated or co-stimulated with anti-CD3/antiCD28 in the absence or the presence of 10 mM NALA or 100 nM rapamycin as described in Methods. In **B**, cell viability measured by the MTT assay after 24 h of co-stimulation and treatments with NALA or rapamycin. In **C-D**, cell divisions determined after staining with CFSE and following 48 h (**C**) and 72 h (**D**) of culturing of unstimulated or co-stimulated cells in the absence or presence of NALA. Representative histograms of division picks and average of divided cells are shown. In all panels, data are average + SEM and represent between 2-3 independent experiments with n>3 mouse spleens pooled/variant/experiment. Statistical significance as determined by a two-tailed Student's t-test: *p<0.05, **p<0.01, ***p<0.001 or ns= no significance.

Supplementary Fig. 2

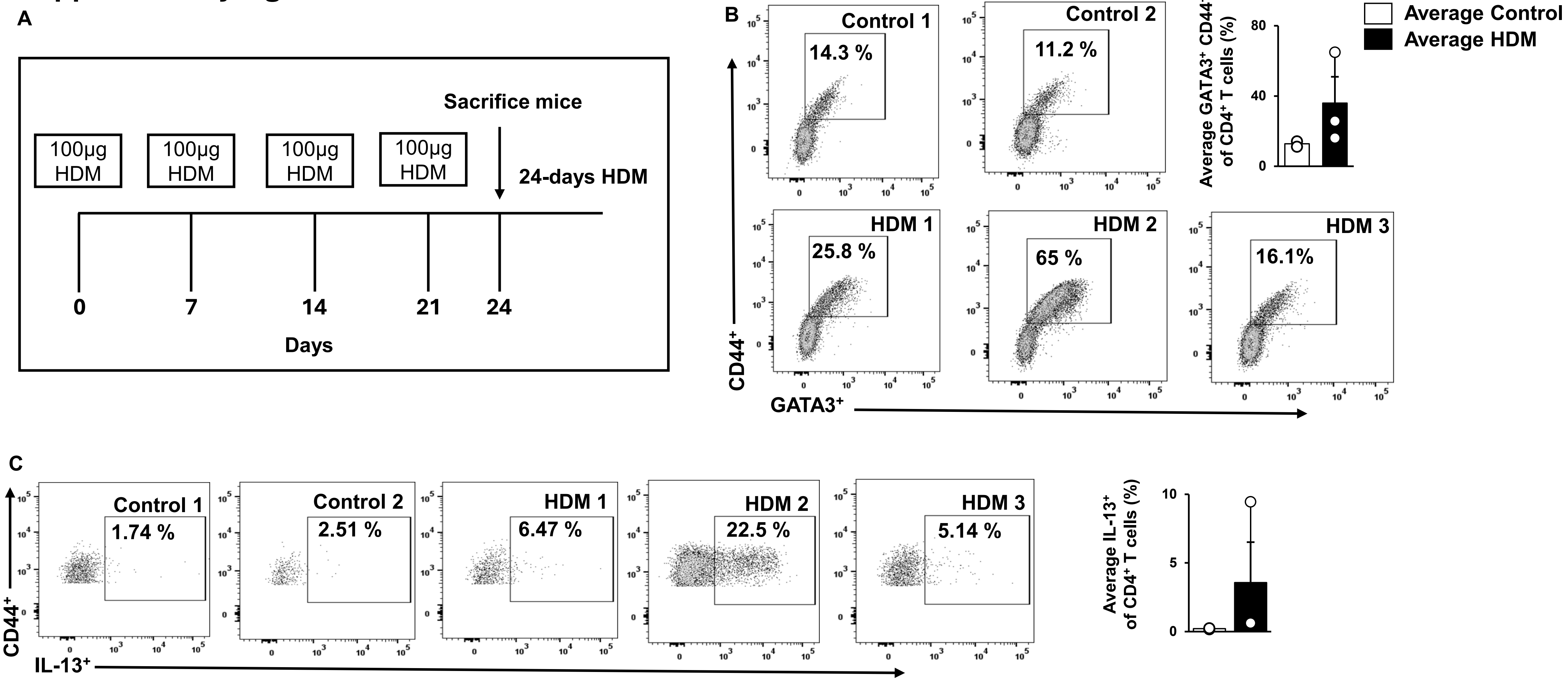

Supplementary Figure 2. House dust mites (HDM) experimental design and pilot testing. (A) Experimental design. **(B-C)** Pilot HDM experiment with WT mice receiving intranasal administrations of 100 µg HDM extract in saline solution (HDM, n=3) or saline only (Control, n=2) once weekly for four consecutive weeks. Representative flow plots along with averaged bar graphs showing GATA3 expression **(B)** and production of IL-13 **(C)** in T cells from lung single-cell suspensions of HDM-treated and control mice. Plots show events gated on viable, $CD3^+CD4^+$ T cells for cytokine staining and on viable, $CD3^+CD4^+Foxp3^-$ T cells for GATA3 staining.

Supplementary Fig. 3

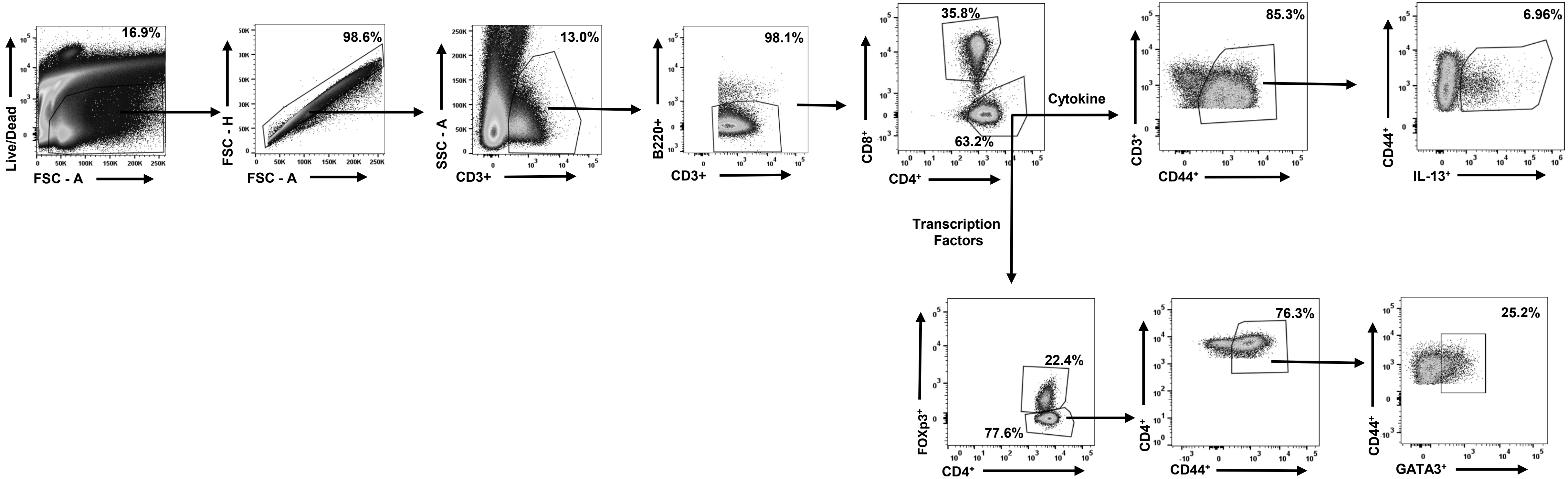

Supplementary Figure 3. Gating strategy used to identify CD4+ Th2 populations in lung single-cell suspensions of HDM-treated, or saline-treated, WT mice or HDM-treated –T-BCATm$^{fl/fl}$ and T-BCATm^{KO} mice.

Supplementary Fig. 4

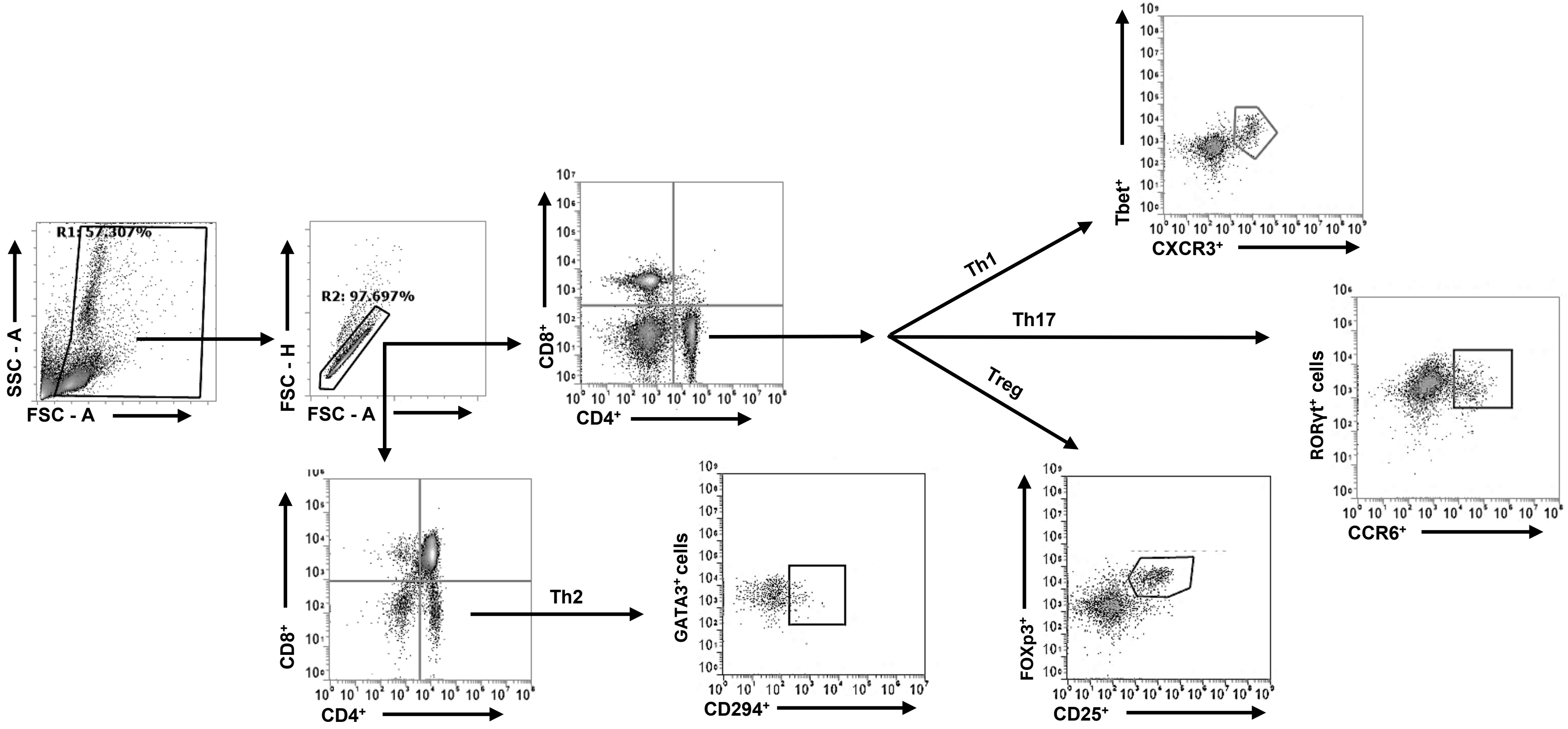

Supplementary Figure 4. Gating strategy used to identify CD4⁺ T cell populations (Th1, Th17 and Tregs) in spleens and lymph nodes or thymus (Th2 only) isolated from naïve or OVA-induced T-BCATc^{KO} and T-BCATm^{KO} mice and their respective controls.

Supplementary Fig. 5

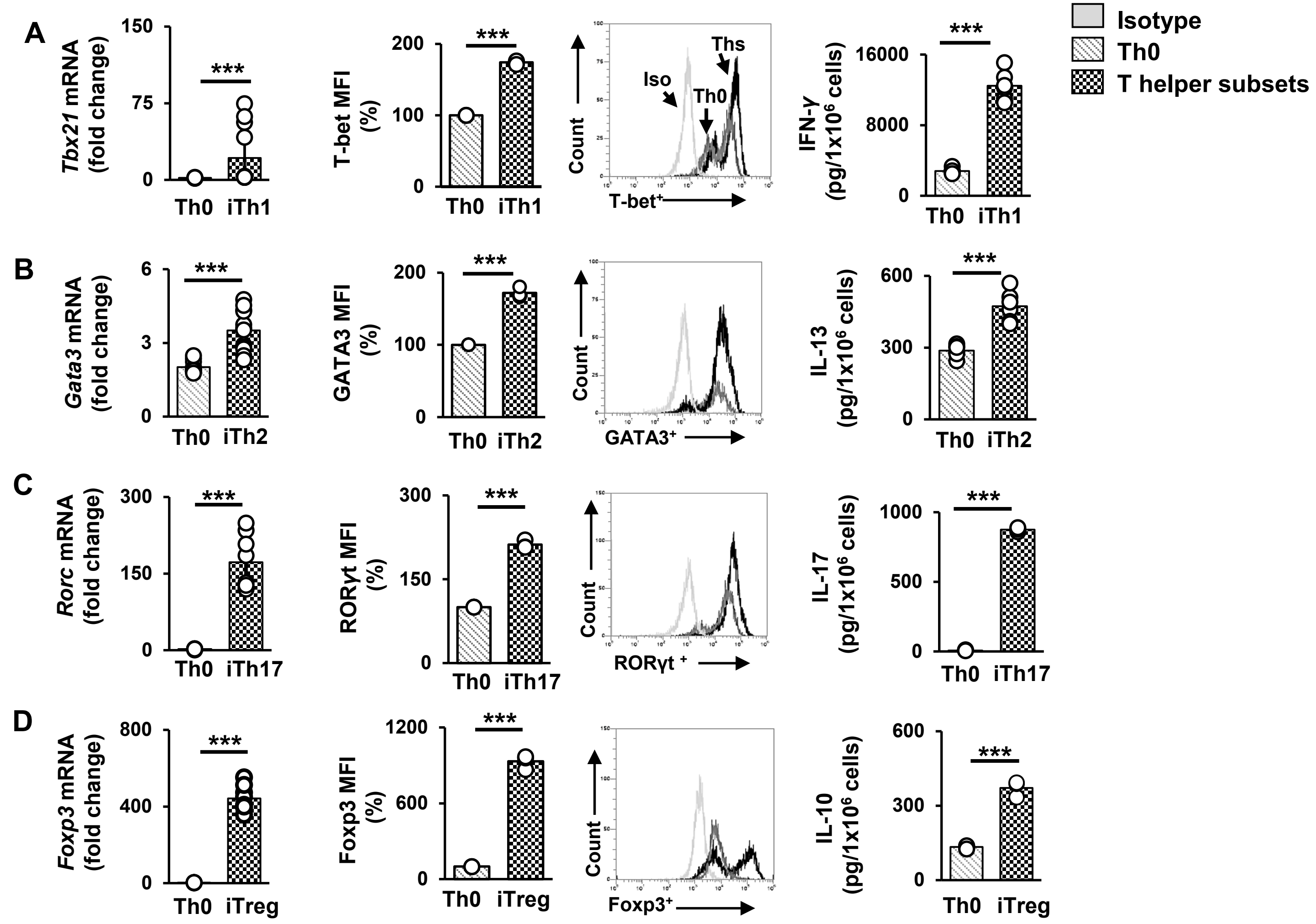

Supplementary Figure 5. Validation of *in vitro* differentiation of CD4⁺ T cells. CD4⁺ T cells, isolated from mouse spleens, underwent *in vitro* differentiation to iTh1 **(A)**, iTh2 **(B)**, iTh17 **(C)**, and iTreg **(D)** cells. Lineage commitment was confirmed with increased gene and protein expression of lineage-specific transcription factors (T-bet, GATA3, RORγt, and Foxp3, respectively) and functional secretion of lineage cytokines (IFN-*γ,* IL-13, IL-17, and IL-10, respectively*)* for each CD4⁺ T cell subset. In all panels, data represent between 2-3 independent experiments with n$\geq$3 mouse spleens pooled/experiment. Representative flow charts and average $\pm$ SEM of mixed sex. Statistical significance as determined by a two-tailed Student's t-test: ***p<0.001. Legend for flow charts, Iso, isotope (light gray); Th0, undifferentiated cells (dark gray); Ths, T helper subset (black).

Supplementary Fig. 6

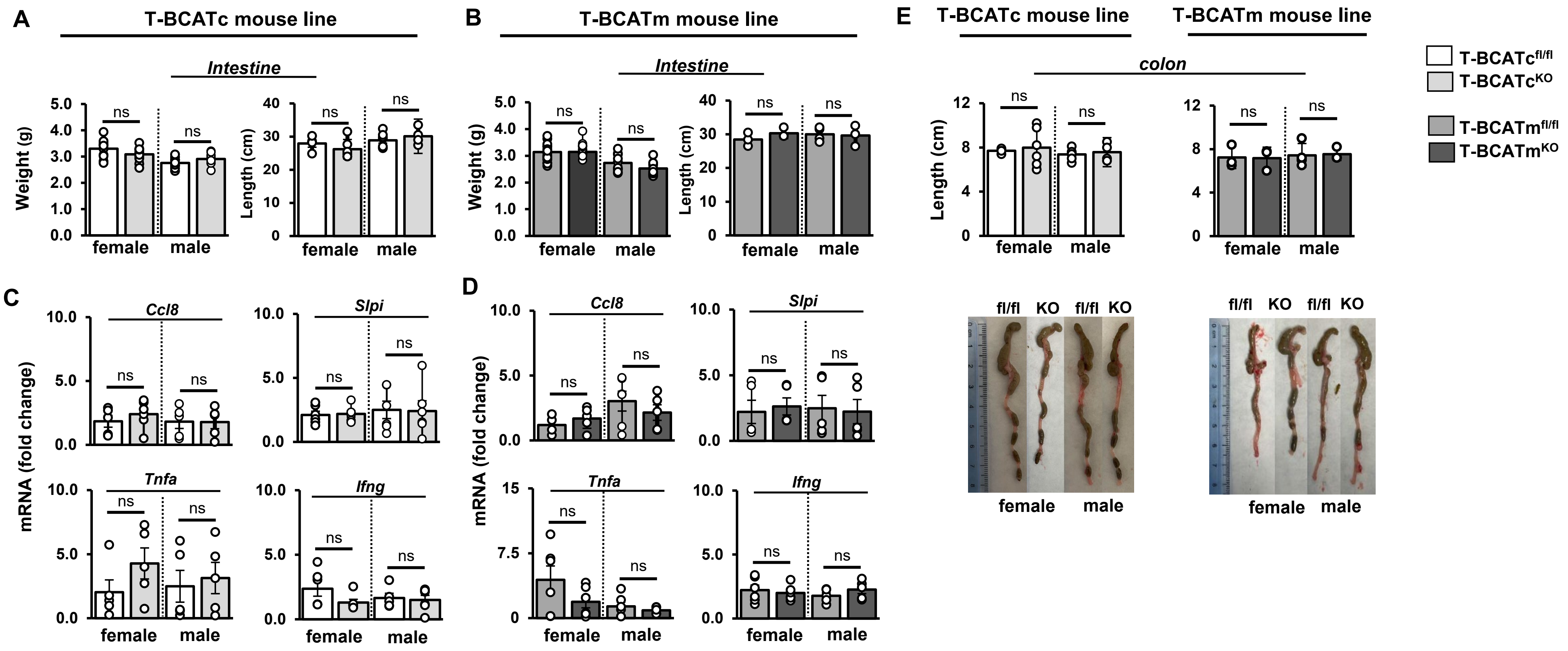

Supplementary Figure 6. A loss of *Bcat1 or Bcat2* expression from mouse T cells does not manifest in an inflammatory phenotype in intestines or colons. Total intestine weights and small intestine lengths **(A-B)**, mRNA expression of small intestine inflammatory markers **(C-D)**, and colon lengths with representative images **(E)** were measured in naïve T-BCATc^{KO} and T-BCATm^{KO} mice and compared to aged-matched control T-BCATc$^{fl/fl}$ and T-BCATm$^{fl/fl}$ mice, separated by sex. Total intestine weights were standardized to a 25 g body weight. In all panels, data represent average $\pm$ SEM, n=6 mice/sex/genotype. Statistical significance was determined by a two-tailed Student's t-test: ns: no significance. *Ccl8:* chemokine ligand 8; *Slpi*: secretory leukocyte protease inhibitor; *Tnfa*: tumor necrosis factor alpha; *Ifng*: interferon gamma.

Supplementary Fig. 7

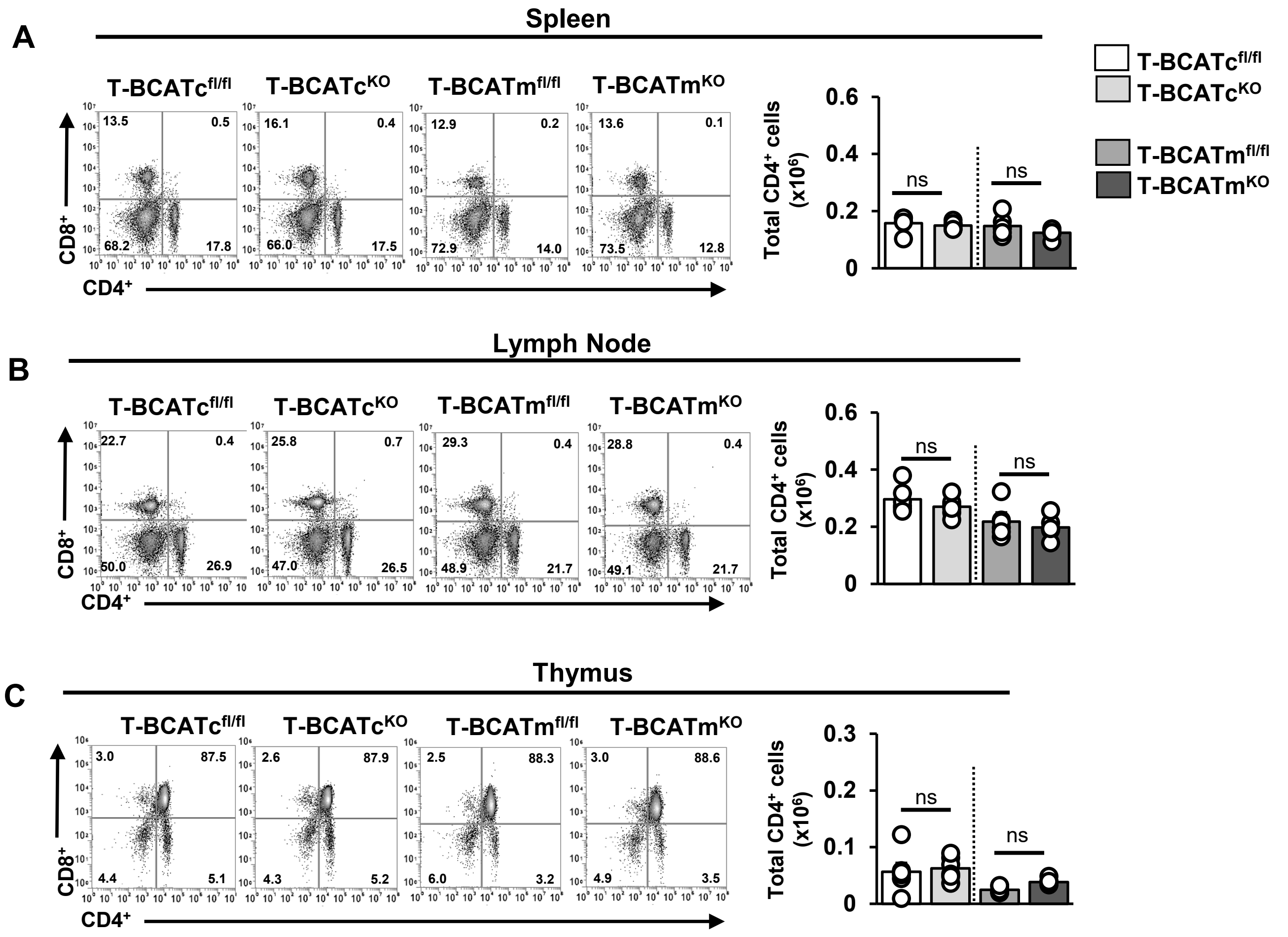

Supplementary Figure 7. **A loss of *Bcat1* or *Bcat2* does not influence CD4+ T cell populations in naïve mice.** Spleens (**A**), lymph nodes (**B**), and thymus (**C**), were isolated from naïve T-BCATc^{KO} and T-BCATm^{KO} mice, homogenized and stained to quantify total CD4+ T cells as compared to naïve, aged-matched T-BCATc$^{fl/fl}$ and T-BCATm$^{fl/fl}$ control mice. Representative flow plots are shown along with average data for each variant. In all panels, data represent average ± SEM of mixed sex with n=6-12 mice/genotype/subset. Statistical significance as determined by a two-tailed Student's t-test: *p<0.05, ns: no significant difference.

Supplementary Fig. 8

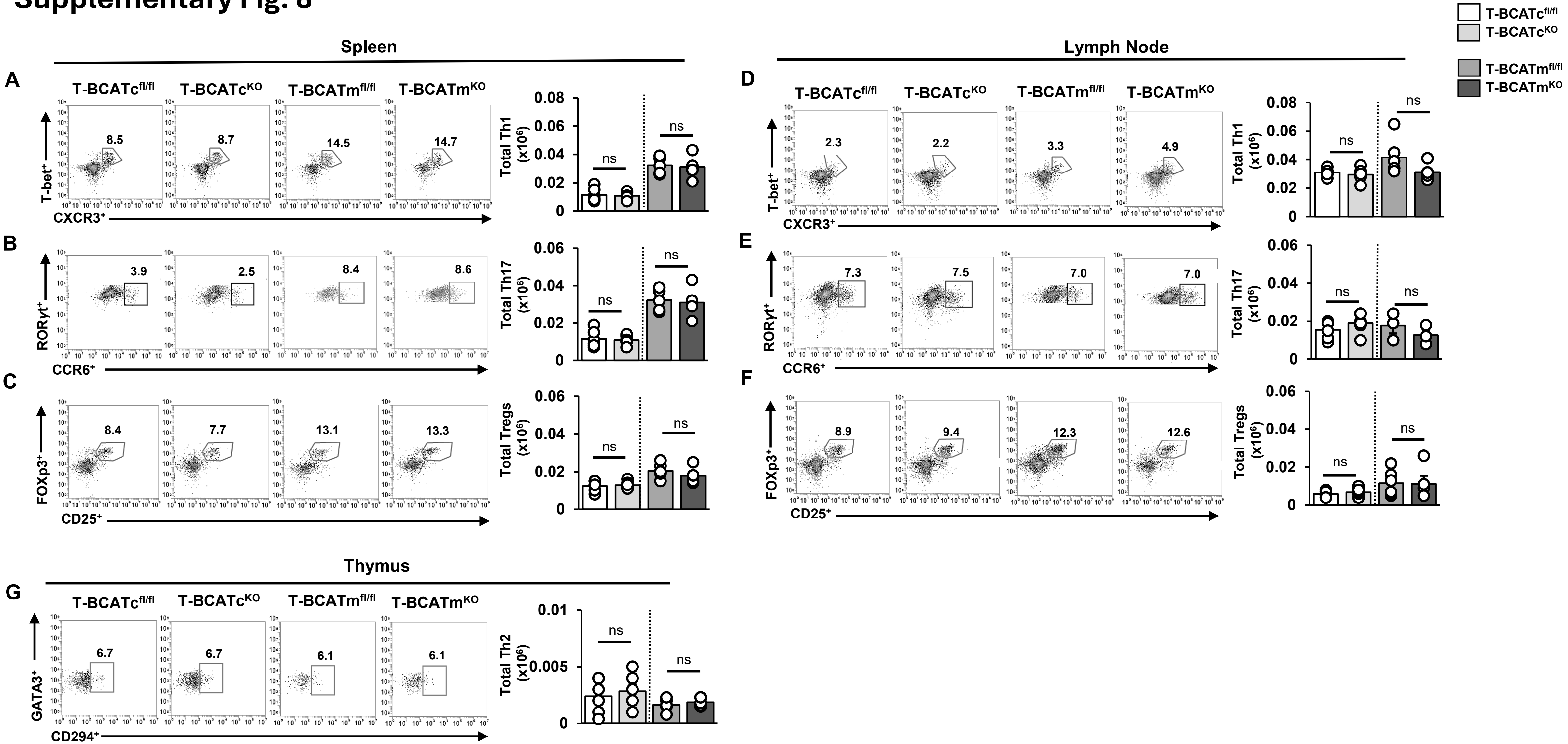

Supplementary Figure 8. **A loss of *Bcat1* or *Bcat2* does not influence CD4⁺ T cell subset populations in naïve mice.** Spleens (**A**,**B**,**C**), lymph nodes (**D**,**E**,**F**), and thymuses (**G**), were isolated from naïve T-BCATc^{KO} and T-BCATm^{KO} mice, homogenized and stained to quantify total Th1, Th17, and Treg cells (spleens, lymph nodes) or Th2 (thymus) as compared to naïve, aged-matched T-BCATc$^{fl/fl}$ and T-BCATm$^{fl/fl}$ mice. Representative flow plots of the co-expression of subset-related surface markers and transcription factors are shown, along with average data for each variant. In all panels, data represent average ± SEM of mixed sex with n=6-12 mice/genotype/subset. Statistical significance as determined by a two-tailed Student's t-test: *p<0.05, ns: no significant difference.

Supplementary Fig. 9

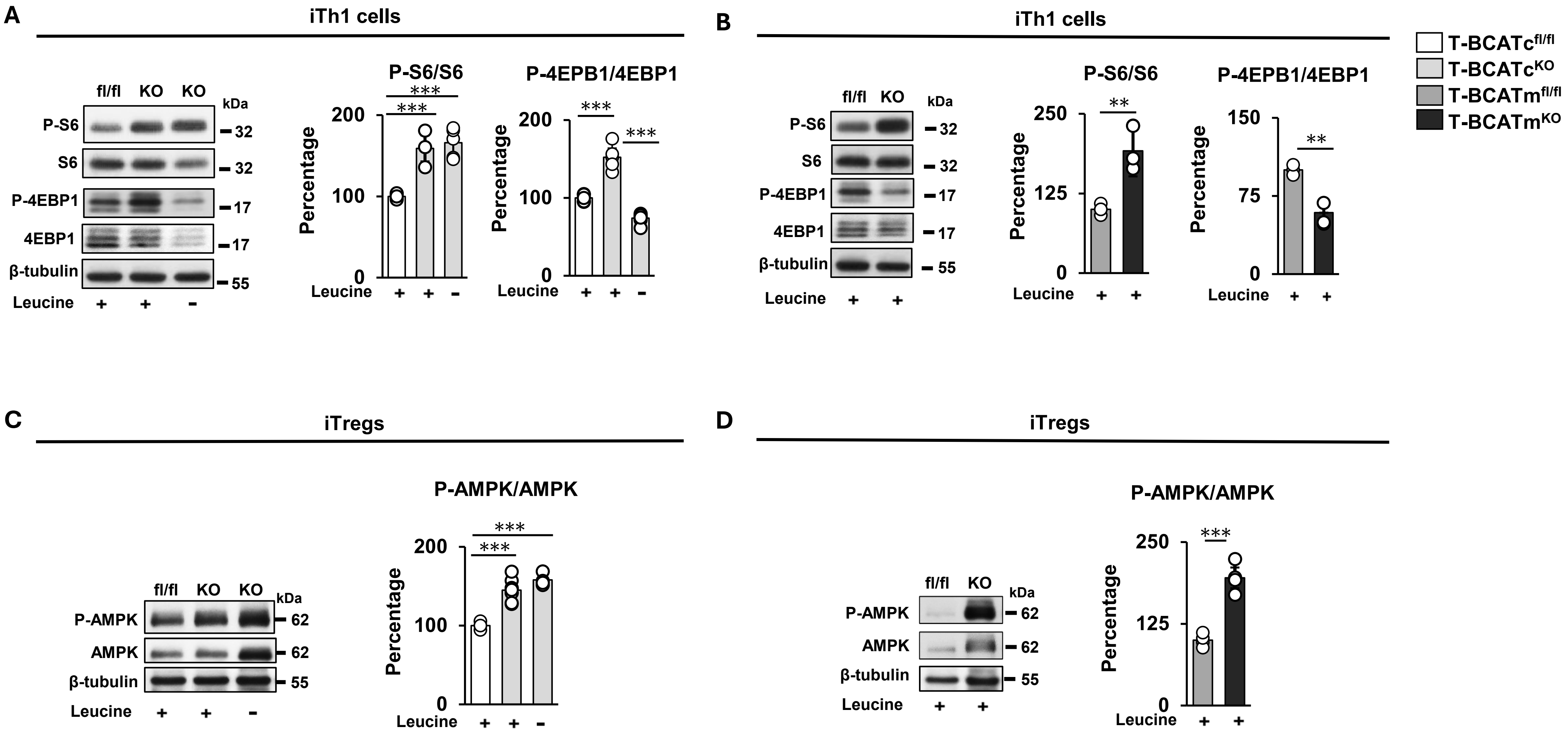

Supplementary Figure 9. A-D. CD4$^+$ T cells from T-BCATc^{KO} (KO) and T-BCATm^{KO} (KO) mice and respective floxed (fl/fl) controls *were in vitro* differentiated into Th1 (**A-B)** and Tregs (**C-D**) in complete RPMI1640 medium (indicated as Leucine "+"). Some of the BCATc-deficient-Th1 and Tregs were incubated in leucine –free medium (indicated as Leucine "-") as described in Methods. In **A-B**, protein expression and phosphorylation of S6 and 4EBP-1 as assessed by western botting. In **C-D**, protein expression of AMPK and its phosphorylation state. In all panels, β-tubulin was used as a loading control. Image J was used to quantify the relative band intensity of P-S6 and S6, P-4EBP-1 and 4EBP-1, and P-AMPK and AMPK and their ratios are presented as percentage of respective T cells from floxed mice. The Western blot images were representative biological replicates from 6 mice/variant (T-BCATc colony) or 4 mice/variant (T-BCATm colony). Average ± SEM of mixed sex. Statistical significance as determined by a two-tailed Student's t-test: *p<0.05, **p<0.01, ***p<0.001 or ns= no significance.
